# Equivalent linearization modeling and experimental validation of natural frequency and vibration isolation performance in metal braided hoses

**DOI:** 10.1371/journal.pone.0361869

**Published:** 2026-11-03

**Authors:** Dacheng Huang, Yue Liu, Dongqin Li, Jianrun Zhang

**Affiliations:** 1 School of Naval Architecture and Intelligent Manufacturing, Jiangsu Maritime Institute, Nanjing, China; 2 School of Mechanical Engineering, Southeast University, Nanjing, China; Ferdowsi University of Mashhad, IRAN, ISLAMIC REPUBLIC OF

## Abstract

Metal braided hoses are widely used in the petroleum industry, marine engineering, aerospace, and other fields for their excellent displacement compensation and vibration damping. Stiffness properties have attracted most of the research so far, while the dynamic behavior of the hose remains comparatively understudied. This paper therefore establishes single-degree-of-freedom axial and bending dynamic models based on the Bouc-Wen hysteresis model and linearizes the nonlinear equations through energy-equivalent linearization, using the energy dissipated by the hysteresis loop. The linearized equations are solved analytically and validated against frequency sweep tests. The model predicts axial natural frequencies of 11.91 Hz and 19.60 Hz for the first two modes and 13.2 Hz for the first bending mode, all close to the measured values, so the equivalent linearized model can be considered reliable for engineering calculation in the low-frequency range. Experiments on hoses with different diameters, lengths, and braiding-layer numbers further show that the bellows size mainly determines the hose stiffness: larger bellows are stiffer but damp less, and adding braiding layers improves damping only until the accompanying increase in stiffness weakens isolation. In the measurements, the two-layer DN25 hose reached the highest axial vibration level difference of 12.83 dB, the longer DN25 hose reached 25.46 dB in bending, and the stiffer DN50 hose isolated much less, at 7.27 dB axially. These quantitative results provide concrete guidance for the design and application of metal braided hoses.

## 1. Introduction

As a core flexible compensation component in piping systems, metal braided hoses are mainly composed of metal bellows, braided mesh sleeves, and end fittings. With excellent flexible deformability, they can not only realize flexible connections between pipelines, but also effectively compensate for displacement deviations in piping systems under complex operating conditions. With the continuous advancement of relevant research, the functional properties of metal braided hoses have been further explored. In addition to displacement compensation, they also exhibit excellent performance in vibration suppression and noise attenuation. At present, metal braided hoses are widely used in pipeline engineering, mining machinery, petroleum equipment, nuclear power facilities, marine and aerospace engineering, and other fields [1–5].

Concerning the vibration-damping performance of bellows, Henan University of Science and Technology has carried out relatively systematic and in-depth investigations. Lu Yongchao [6] studied the equivalent damping characteristics of multi-layer bellows with interlayer damping. The results indicated that the loss factor of multi-layer bellows is closely related to the properties of viscoelastic damping materials and the structural parameters of the bellows themselves. Zhao Pei [7] focused on analyzing the influences of structural parameters of multi-layer bellows and properties of viscoelastic materials on their dynamic stiffness. It is found that the pitch, wall thickness, root diameter of bellows, and elastic modulus of viscoelastic materials are all positively correlated with the dynamic stiffness of bellows, while the dynamic stiffness is negatively correlated with the number of convolutions. In addition, the thickness of damping materials has a relatively limited effect on the dynamic stiffness of bellows. Li Changzhi [8] investigated the vibration isolation performance of multi-layer bellows. The results showed that the relationship between the vibration-damping effect of bellows and their structural parameters, as well as damping layer thickness, is not completely consistent under different excitation frequencies. Liu Yonggang [9] studied the stiffness and damping properties of metal bellows in piping systems. Numerical simulations clarified the inherent correlations among the loss factor, axial stiffness, and structural parameters of metal bellows, and experiments revealed the evolution laws of stiffness and damping with water pressure in piping systems. The findings provide theoretical support and engineering guidance for the structural design of multi-layer bellows in follow-up studies. Radhakrishna [10] explored the influence of elastic constraints at both ends on the axial natural frequency of bellows and established a corresponding theoretical model. The numerical results are in good agreement with the experimental data reported by Li [11]. Xiong Yonghua [12] analyzed key dynamic performance parameters such as natural frequency and mechanical impedance of DN200 metal bellows, which lays a foundation for the engineering application of this type of bellows. Wang [13] used a combination of finite element analysis and experiments to verify the vibration characteristics of the S-shaped corrugated tube. Wang and Chen employed the method of spiral beam modeling to study the axial mechanical properties of metal corrugated tubes [13,14].

In recent years, researchers have increasingly used friction to design damping, energy-dissipating vibration isolation devices, such as wire rope and metal rubber isolators. Zou [15] investigated the hysteretic mechanical behavior of a spring‑metal wire mesh rubber hybrid isolator and identified the damping mechanism of metal rubber in vibration mitigation systems. Guo [16] used the Galerkin method to examine the effects of grounded inerter dampers on the stability and nonlinear dynamic responses of cantilevered fluid-conveying pipes. Li [17] studied the dynamic response and vibration suppression of such pipes under periodic excitation, achieving precise system prediction via a fluid‑structure interaction model. Aloschi [18] effectively attenuated pipeline vibrations through optimal internal damping and vibratory impact system design. Mamaghani [19] suppressed flow-induced pipeline vibrations using a nonlinear energy sink (NES). Bi and Hao [20] proposed an innovative pipe‑in‑pipe system for subsea vibration control, simplifying it as a tuned mass damper (TMD) for high-performance passive damping. Zhang [21] analyzed piping damping performance with piezoelectric active constrained layer materials and proposed an optimized placement scheme. Gong [22] developed a magnetorheological damping clamp and realized active pipeline vibration control via a fuzzy PID algorithm.

Despite extensive research on the mechanical properties, damping modeling, and dynamic response of metal braided hoses and their components, significant gaps remain. First, stiffness analyses rely heavily on bellows theoretical models, with insufficient investigation into braided metal mesh stiffness and its coupling with bellows, preventing accurate characterization of overall stiffness nonlinearity. Second, systematic studies on metal braided hose dynamic response and damping performance are limited; most focus on statics, with incomplete analyses of transient response, frequency behavior, and damping mechanisms. Furthermore, foreign studies often focus on specific operating conditions, lacking systematic exploration of relationships between structural parameters, dynamic performance, and vibration reduction effectiveness, failing to meet diverse engineering needs.

To address these gaps, this work thoroughly investigates the metal braided hose dynamic response and vibration isolation performance. The research approach is as follows: (1) systematically analyze the structural features of metal bellows and braided mesh, and their coupling mechanisms, then develop an accurate equivalent dynamic model using existing theories and experimental data; (2) apply analytical methods to solve and analyze hose dynamic response, revealing vibration characteristics and trends; (3) experimentally evaluate vibration isolation performance of hoses with varying structural parameters to validate the model and quantify parameter effects on damping. This study provides robust theoretical support and technical guidance for metal braided hose design, manufacturing optimization, and engineering applications, filling research gaps and advancing dynamic characteristic and vibration control research for metal braided hoses.

The remaining part of this article is arranged as follows: Section 2 elaborates on the dynamic equations of the metal braided hose, including the establishment of the dynamic model based on the Bouc-Wen model and the linearization of the model using the energy equivalent method. Section 3 introduces the dynamic experiment process of the metal braided hose, presenting the overall design and samples of the experiment. Section 4 presents the results of the axial and bending vibration isolation experiments of the metal braided hose, evaluating the vibration isolation performance of the metal braided hose. Section 5 explains the results, compare with previous studies, clarify the limitations of this research, and provide engineering implications, and Section 6 summarizes the main conclusions and looks forward to future work.

## 2. Dynamics model of metal braided hose

### 2.1. Hysteresis model of metal braided hose

Since the axial force and displacement of the metal braided hose do not exhibit a linear relationship, based on previous research [23,24], the relationship between the axial force and displacement of the metal braided hose can be expressed as:


$$\begin{array}{c} F(t)=\sum_{\text{0}}^{\tilde{N}}k_{Ei}x^{i}+Z(t)\sum_{\text{0}}^{\tilde{M}}k_{Aj}x^{j} \end{array}$$ (1)

$$\begin{array}{c} \dot{Z}(t)=\rho \dot{x}(t)\left(\text{1}-\left[\sigma sgn\left(\dot{x}(t)\right)sgn\left(Z(t)\right)-\sigma +\text{1}\right]{\left|Z(t)\right|}^{u}\right) \end{array}$$ (2)

Where F(t) is the total restoring force, x(t) is the input excitation displacement, and Z(t) is the pure hysteretic restoring force. $\dot{x}(t)$ and $\dot{Z}(t)$ denote the first derivatives of x(t) and Z(t) with respect to time t, respectively. Here u≥0,σ≥1/2, ρ≥0,$k_{Ei}$ is the polynomial coefficient of the nonlinear elastic term, and N is the order of the nonlinear elastic polynomial; $k_{Aj}$ is the polynomial coefficient of the nonlinear amplification factor, and M is the order of the nonlinear amplification factor polynomial.

### 2.2. The motion equation of the metal braided hose system

The equation of motion for a single-degree-of-freedom system under forced vibration can be written as:


$$\begin{array}{c} m\ddot{x}+c\dot{x}+kx=P_{\text{0}}sin(\omega t) \end{array}$$ (3)

One end of the system is fixed, and the other end is subjected to an external force $F=P_{\text{0}}\text{sin}(\omega t)$, where x is the displacement under the external force. The metal braided hose is equivalent to a single-degree-of-freedom spring-damping system, as shown in Fig 1. The equation of motion under harmonic excitation is given by Eq. (3). Based on the hysteretic model of the metal braided hose, its equation of motion under excitation force can be expressed as:

**Fig 1:**
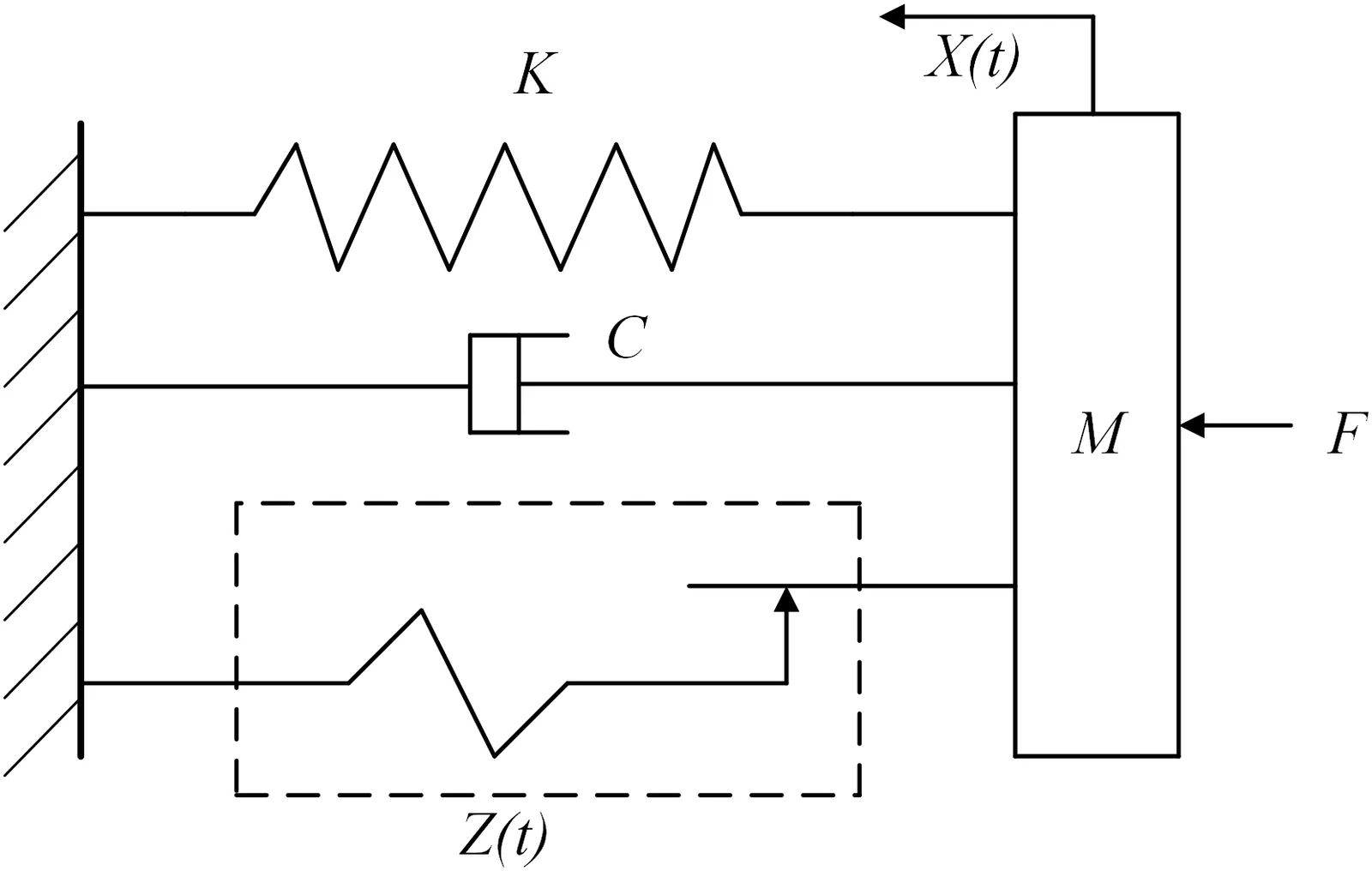
Vibration mechanics model of a metal braided hose.


$$\begin{array}{c} m\ddot{x}+\sum_{\text{0}}^{\tilde{N}}k_{Ei}x^{i}+Z(t)\sum_{\text{0}}^{\tilde{M}}k_{Aj}x^{j}=P_{\text{0}}sin(\omega t) \end{array}$$ (4)

$$\begin{array}{c} \dot{Z}(t)=\rho \dot{x}(t)\left(\text{1}-\left[\sigma sgn\left(\dot{x}(t)\right)sgn\left(Z(t)\right)-\sigma +\text{1}\right]{\left|Z(t)\right|}^{u}\right) \end{array}$$ (5)

where m is the mass of the hose, $P_{\text{0}}$ is the amplitude of the excitation force, $k_{E}$ and $k_{A}$ are the polynomial coefficients of the nonlinear elastic term and the nonlinear amplification factor, respectively, and Z(t) is the hysteretic force.

To avoid calculation errors introduced by unit issues, the system model is nondimensionalized [25]. The displacement scale X and time scale τ are introduced.


$$\begin{array}{c} \lambda =\frac{x}{X} \end{array}$$ (6)

$$\begin{array}{c} \tau =\omega t \end{array}$$ (7)

Substituting Eqs. (6) and (7) into Eqs. (4) and (5), the nondimensional equation of motion for the nonlinear system of the metal braided hose can be obtained as:


$$\begin{array}{c} \ddot{\lambda }(\tau )+\sum_{\text{0}}^{\tilde{N}}\beta_{Ei}\lambda^{i}(\tau )+Z(\tau )\sum_{\text{0}}^{\tilde{M}}\beta_{Aj}\lambda^{j}(\tau )=\tilde{P_{\text{0}}}sin(\tau ) \end{array}$$ (8)

$$\begin{array}{c} \dot{Z}(\tau )=\rho_{X}\dot{\lambda }(\tau )\left(\text{1}-\left[\sigma sgn\left(\dot{\lambda }(\tau )\right)sgn\left(Z(\tau )\right)-\sigma +\text{1}\right]{\left|Z(\tau )\right|}^{u}\right) \end{array}$$ (9)

In which, $\ddot{\lambda }(\tau )$, $\dot{\lambda }(\tau )$ and $\dot{Z}(\tau )$ are the derivatives with respect to time τ, respectively. $\ddot{\lambda }(\tau )$ is the second derivative of dimensionless displacement, $\dot{\lambda }(\tau )$ is the first derivative of the dimensionless displacement, and $\dot{Z}(\tau )$ represents the derivative of the hysteresis force with respect to time.


$$\begin{array}{c} \beta_{Ei}=\frac{X^{i-\text{1}}k_{Ei}}{m\omega^{\text{2}}} \end{array}$$ (10)

$$\begin{array}{c} \beta_{Aj}=\frac{X^{j-\text{1}}k_{Aj}}{m\omega^{\text{2}}} \end{array}$$ (11)

$$\begin{array}{c} \rho_{X}=X\rho \end{array}$$ (12)

$$\begin{array}{c} \tilde{P_{\text{0}}}=\frac{P_{\text{0}}}{m\cdot X\cdot \omega^{\text{2}}} \end{array}$$ (13)

In which, $\beta_{Ei}$ is the dimensionless nonlinear elastic polynomial coefficient, $\beta_{Aj}$ is the dimensionless nonlinear amplification factor polynomial coefficient, $\rho_{X}$ is the dimensionless hysteresis parameter, $\tilde{P_{\text{0}}}$ is the dimensionless excitation force. m is the mass, ω is the frequency, X is the displacement scale.

### 2.3. Energy dissipation of the hysteresis model

In the dynamic response of a metal braided hose, the hysteretic force causes energy dissipation, so the energy generated by the hysteretic force needs to be considered. Fig 2 shows the normalized Bouc–Wen hysteresis curve. The area enclosed by the hysteresis curve ABCD represents the energy dissipation of the metal braided hose within one cycle, where points A and C are the extreme points of the input displacement, and points B and D are the points where the hysteretic force Z is zero.

**Fig 2:**
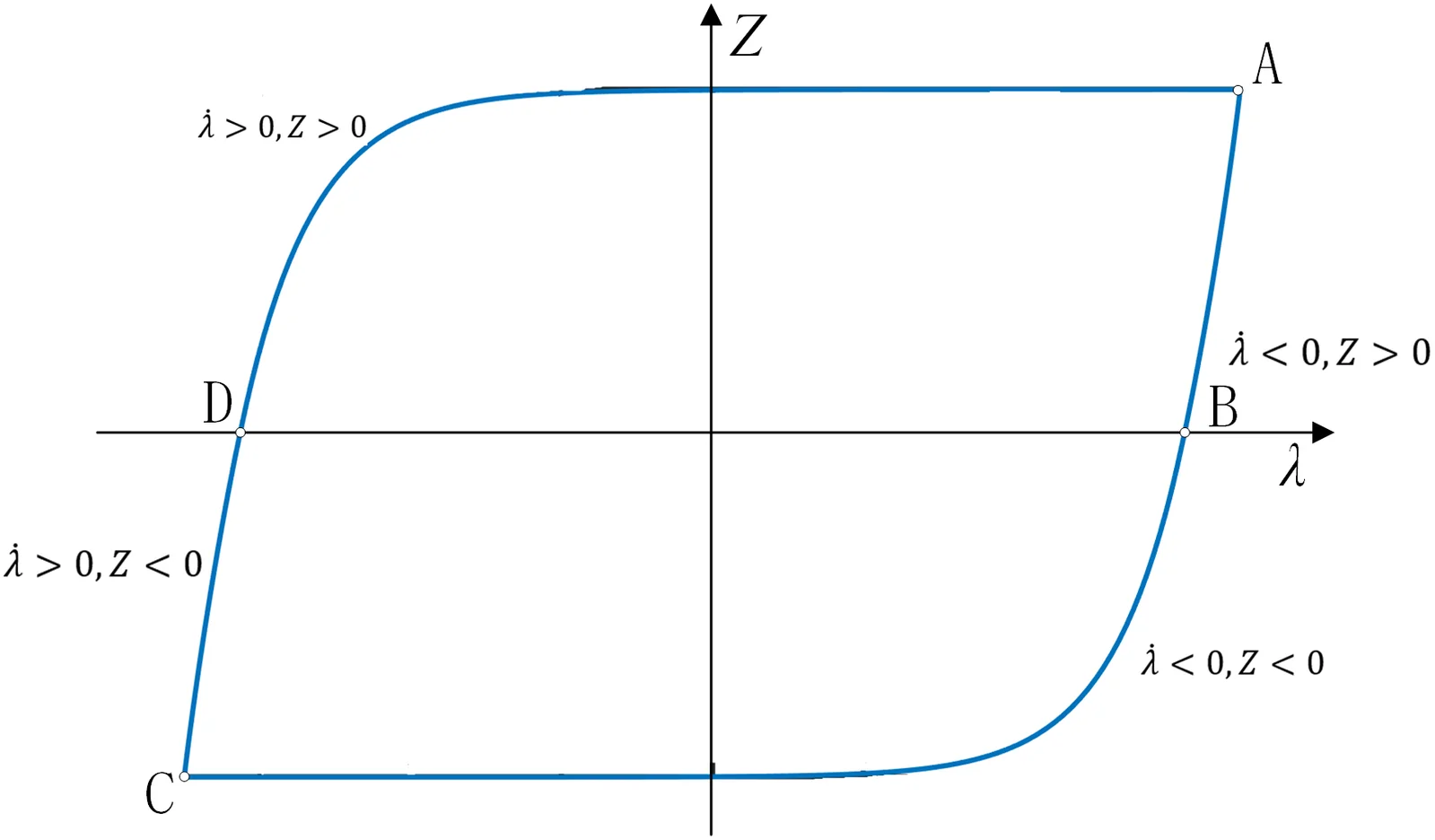
Normalized Bouc-Wen.

According to the directions of $\dot{u}$ and Z, the Bouc–Wen curve can be divided into four segments: AB, BC, CD, and DA. The core criterion is the sign of $\dot{\lambda }$ and Z: $\dot{\lambda }$ and Z have the same sign in segments DA and BC, and opposite signs in segments AB and CD. The hysteretic force model in Eq. (9) is rewritten as a relation between $\dot{\lambda }$ and Z:


$$\begin{array}{c} \frac{d\lambda }{dZ}=\frac{\text{1}}{\rho_{X}\left(\text{1}-\left[\sigma sgn\left(\dot{\lambda }(\tau )\right)sgn\left(Z(\tau )\right)-\sigma +\text{1}\right]{\left|Z(\tau )\right|}^{u}\right)} \end{array}$$ (14)

Among them, σ, u and $\rho_{X}$ is dimensionless hysteresis parameter, Z(τ) is the dimensionless hysteretic force, $\dot{\lambda }(\tau )$ The first derivative of the dimensionless displacement.

According to the sign relationship between $\dot{u}$ and Z, Eq. (14) can be further expressed as:


$$\begin{array}{c} \frac{d\lambda }{dZ}=\left\{ \begin{array}{c} \frac{\text{1}}{\rho_{X}\left(\text{1}-{\left|Z(\tau )\right|}^{u}\right)}\;\dot{\lambda }\text{ and }Z\text{ have the same sign}\\ \frac{\text{1}}{\rho_{X}\left(\text{1}-[\text{1}-\text{2}\sigma ]{\left|Z(\tau )\right|}^{u}\right)}\;\dot{\lambda }\text{ and }Z\text{ have the opposite sign} \end{array}\right. \end{array}$$ (15)

According to research by Charalampakis [26], the Gaussian hypergeometric function can represent the solution of the differential equation. Based on this, this paper adopts this function to express the analytical solution of Eq. (15):


$$\begin{array}{c} \lambda (Z)=\left\{ \begin{array}{c} @l{\frac{Z}{\rho_{X}}}_{\text{2}}F_{\text{1}}\left(\text{1},\frac{\text{1}}{u};\text{1}+\frac{\text{1}}{u};{\left|Z(\tau )\right|}^{u}\right)+C_{\text{1}}\;\dot{\lambda }\;and\;Z\;have\;the\;same\;sign\\ {\frac{Z}{\rho_{X}}}_{\text{2}}F_{\text{1}}\left(\text{1},\frac{\text{1}}{u};\text{1}+\frac{\text{1}}{u};(\text{1}-\text{2}\sigma ){\left|Z(\tau )\right|}^{u}\right)+C_{\text{2}}\;\dot{\lambda }\;and\;Z\;have\;the\;opposite\;sign \end{array}\right. \end{array}$$ (16)

where $C_{\text{1}}$ and $C_{\text{2}}$ are integration constants determined by initial conditions, and $\backslash {(}_{\text{2}}F_{\text{1}}$ denotes the Gaussian hypergeometric function.

As shown in Fig 3, the area enclosed by the hysteresis loop ABCD can be calculated using the area subtraction method, taking advantage of the centrosymmetric property of the normalized Bouc‑Wen model about the origin. First, the area of AA’CC’ is obtained, followed by the area of ADCC’A. The final hysteresis area is equal to the area of AA’CC’ minus twice the area of ADCC’A. The calculation formula is as follows:

**Fig 3:**
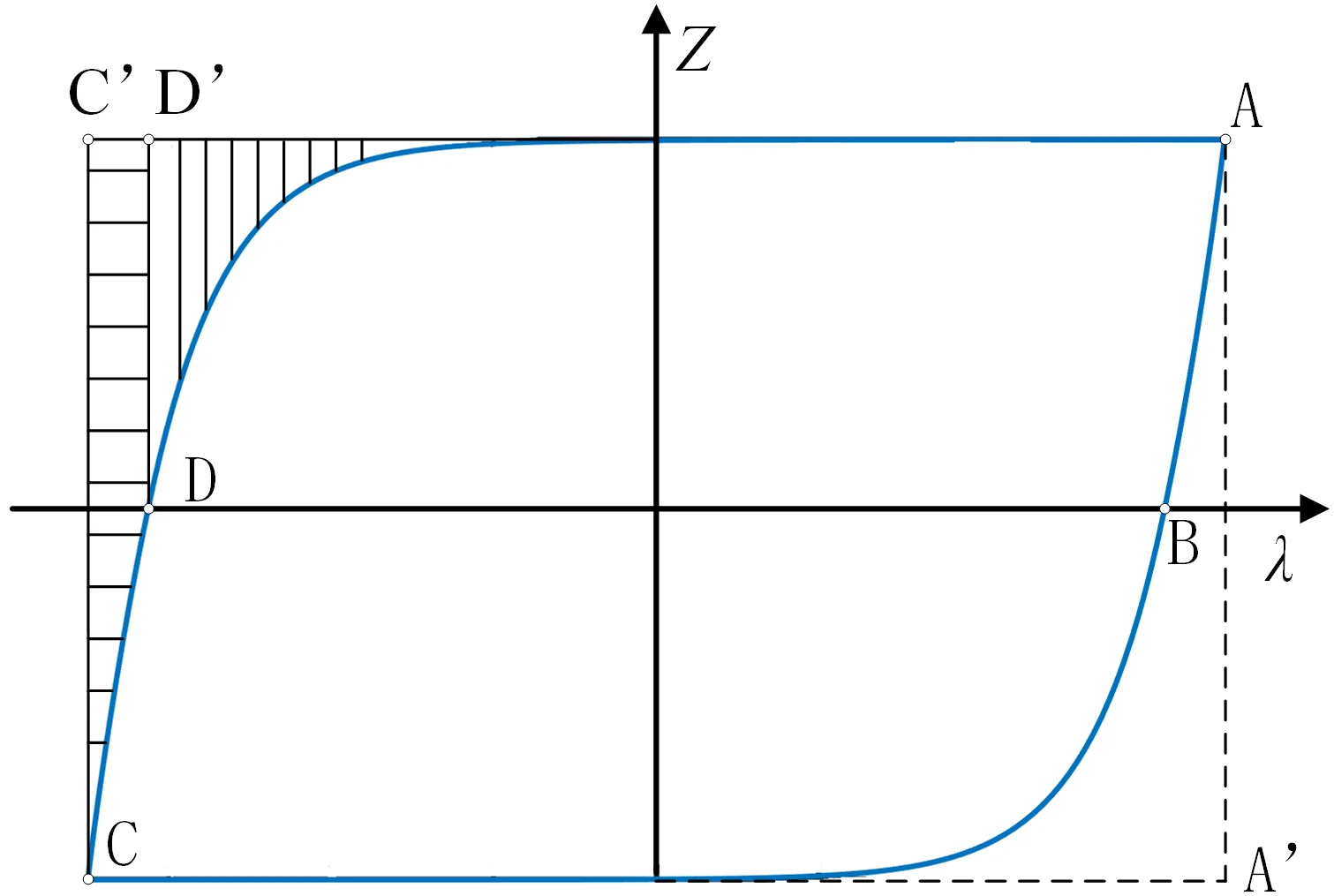
Schematic diagram of Bouc-Wen energy calculation.


$$\begin{array}{c} E=\text{4}\lambda_{A}Z_{A}-\text{2}\left(E_{ADD^{\prime }}+E_{DCC^{\prime }D^{\prime }}\right) \end{array}$$ (17)

Among them, E represents the area of the hysteresis loop ABCD, $\lambda_{A}$ represents the maximum displacement, $Z_{A}$ represents the maximum value of the hysteresis force, $E_{ADD^{\prime }}$ represents the area enclosed by the letters ADD,’ $E_{DCC^{\prime }D^{\prime }}$ represents the area enclosed by the letters DCC'D.’

$E_{ADD^{\prime }}$ and $E_{DCC^{\prime }D^{\prime }}$ can be obtained through integration. Combined with the Gaussian hypergeometric function, their analytical expressions are derived as follows.


$$\begin{array}{c} E_{ADD^{\prime }}={\frac{{Z_{A}}^{\text{2}}}{\rho_{X}}}_{\text{2}}F_{\text{1}}\left(\text{1},\frac{\text{1}}{u};\text{1}+\frac{\text{1}}{u};{Z_{A}}^{u}\right)-{\frac{{Z_{A}}^{\text{3}}}{{\text{2}\rho }_{X}}}_{\text{2}}F_{\text{1}}\left(\text{1},\frac{\text{1}}{u};\text{1}+\frac{\text{1}}{u};{Z_{A}}^{u}\right) \end{array}$$ (18)

$$\begin{array}{c} E_{DCC^{\prime }D^{\prime }}={\frac{{Z_{A}}^{\text{2}}}{\rho_{X}}}_{\text{2}}F_{\text{1}}\left(\text{1},\frac{\text{1}}{u};\text{1}+\frac{\text{1}}{u};(\text{1}-\text{2}\sigma ){Z_{A}}^{u}\right)+{\frac{{Z_{A}}^{\text{3}}}{{\text{2}\rho }_{X}}}_{\text{2}}F_{\text{1}}\left(\text{1},\frac{\text{1}}{u};\text{1}+\frac{\text{1}}{u};(\text{1}-\text{2}\sigma ){Z_{A}}^{u}\right) \end{array}$$ (19)

Once the parameters of the Bouc–Wen model and the maximum amplitude $\lambda_{A}$ are known, they can be substituted into Eq. (17) to calculate the energy dissipation of the model within one cycle.

### 2.4. Equivalent linearization of stiffness and damping of metal braided hoses

The core of equivalent linearization is to use a linear system to characterize a complex nonlinear system. In this paper, the Coulomb damping caused by friction between metal wires was equivalent to viscous damping, and the nonlinear stiffness between the metal bellows and the braided mesh sleeve was replaced by equivalent stiffness. Accordingly, Eq. (8) can be expressed as:


$$\begin{array}{c} \ddot{\lambda }+\left(c_{b}+c_{eq}\right)\dot{\lambda }+k_{eq}\lambda =\tilde{P_{\text{0}}}\text{sin}(\tau ) \end{array}$$ (20)

where $\ddot{\lambda }$ and $\dot{\lambda }$ are the derivatives with respect to the dimensionless time τ; $c_{b}$ is the inherent damping of the bellows, which can be temporarily neglected in this paper; $c_{eq}$ is the equivalent damping coefficient; $k_{eq}$ is the equivalent stiffness coefficient; and $\tilde{P_{\text{0}}}$ is the dimensionless external force.

The axial stiffness of the metal braided hose is described by a polynomial. Under small-displacement conditions, its equivalent stiffness can be approximated by the first-order term of the nonlinear stiffness polynomial, and the equivalent stiffness for the axial dynamic response is thus obtained as:


$$\begin{array}{c} k_{eq}=\beta_{E\text{1}} \end{array}$$ (21)

The bending stiffness of the metal braided hose is inherently linear $K_{y}$, so equivalent linearization is only required for the nonlinear damping in its bending vibration.

Similarly, the nonlinear amplification factor of hysteretic damping for the dynamic response system of the metal braided hose can be approximated by a first-order form under small-displacement vibration:


$$\begin{array}{c} Z(\tau )\sum_{\text{0}}^{\tilde{M}}\beta_{Aj}\lambda^{j}(\tau )\approx Z(\tau )\left(\beta_{A\text{0}}+\beta_{A\text{1}}\lambda^{\text{1}}(\tau )\right) \end{array}$$ (22)

Among the coefficients of the nonlinear hysteretic amplification factor, the value of $\beta_{A\text{0}}$ is much larger than those of other order coefficients. As can be seen from Eq. (11), the system excitation frequency ω>1, so $\beta_{A\text{0}}\gg \beta_{A\text{1}}$ in the first-order approximation Eq. (22) of the hysteretic damping nonlinear amplification factor. Other terms can be ignored in the calculation, and only $\beta_{A\text{0}}$ is retained to simplify the calculation. The simplified dynamic equation is:


$$\begin{array}{c} \ddot{\lambda }+\beta_{E\text{1}}\lambda +\beta_{A\text{0}}Z(\tau )=\tilde{P_{\text{0}}}\text{sin}(\tau ) \end{array}$$ (23)

Degrading Eq. (23) into an equivalent linearization equation yields:


$$\begin{array}{c} \ddot{\lambda }+c_{eq}\dot{\lambda }+k_{eq}\lambda =\tilde{P_{\text{0}}}\text{sin}(\tau ) \end{array}$$ (24)

According to the equivalent damping energy dissipation principle in vibration theory, the energy dissipated by linear damping $E_{c}$ and nonlinear damping $E_{s}$ in one cycle under forced vibration is equal, i.e., $E_{c}=E_{s}$, where $E_{c}$ can be expressed by Eq. (25), and $E_{s}$ can be obtained from the area of the hysteresis curve.


$$\begin{array}{c} E_{c}=\pi c\lambda_{A}^{\text{2}}\omega \end{array}$$ (25)

The equivalent damping of the metal braided hose axial vibration system can be expressed as:


$$\begin{array}{c} c_{eq}=\frac{E_{s}}{\pi \lambda_{A}^{\text{2}}\omega } \end{array}$$ (26)

The main source of energy dissipation in the nonlinear system is $\beta_{A\text{0}}Z(\tau )$. Based on the function modulation of the normalized Bouc-Wen model by Zhao Yu [27], it can be obtained that:


$$\begin{array}{c} E_{s}=\beta_{A\text{0}}E \end{array}$$ (27)

Then the equivalent damping can be expressed as:


$$\begin{array}{c} c_{eq}=\frac{\beta_{A\text{0}}E}{\pi \lambda_{A}^{\text{2}}\omega } \end{array}$$ (28)

where $\beta_{A\text{0}}$ is the first-order coefficient of the hysteretic damping nonlinear amplification factor; E is the dissipated energy calculated by Eq. (17); $\lambda_{A}$ is the vibration amplitude of the damping system; and ω is the excitation frequency.

### 2.5. Numerical solution of metal braided hose

#### 2.5.1. Equivalent approximate solution of metal braided hose.

The steady‑state response displacement of the equivalent linear system under harmonic excitation is:


$$\begin{array}{c} \lambda_{p}(\tau )=\lambda_{A}\text{sin}(\tau -\phi ) \end{array}$$ (29)

where $\lambda_{A}$ is the response amplitude, and ϕ is the phase difference between the input excitation and the output response. τ is the dimensionless time.

Substituting Eq. (29) into Eq. (24) yields:


$$\begin{array}{c} \lambda_{A}\left[\left(k_{eq}-\text{1}\right)\text{sin}(\tau -\phi )+c_{eq}\text{cos}(\tau -\phi )\right]=\tilde{P_{\text{0}}}\text{sin}(\tau ) \end{array}$$ (30)

Among them, $k_{eq}$ is equivalent stiffness, $c_{eq}$ is equivalent damping, $\tilde{P_{\text{0}}}$ is dimensionless external force.

Eq. (30) is simplified using the following trigonometric identities:


$$\begin{array}{c} \left\{ \begin{array}{c} @l\text{sin}(\tau -\phi )=\text{sin}(\tau )\text{cos}(\phi )-\text{cos}(\tau )\text{sin}(\phi )\\ \text{cos}(\tau -\phi )=\text{cos}(\tau )\text{cos}(\phi )+\text{sin}(\tau )\text{sin}(\phi ) \end{array}\right. \end{array}$$ (31)

Equating the coefficients of sin(τ) and cos(τ) on both sides of Eq. (30), we obtain:


$$\begin{array}{c} \left\{ \begin{array}{c} @l\lambda_{A}\left[\left(k_{eq}-\text{1}\right)\text{cos}(\phi )+\beta_{A\text{0}}\text{sin}(\phi )\right]=\tilde{P_{\text{0}}}\\ \lambda_{A}\left[c_{eq}\text{cos}(\phi )-\left(k_{eq}-\text{1}\right)\text{sin}(\phi )\right]=\text{0} \end{array}\right. \end{array}$$ (32)

Then, according to Eq. (23), the amplitude $\lambda_{A}$ and phase ϕ of the system can be derived as:


$$\begin{array}{c} \lambda_{A}=\frac{\tilde{P_{\text{0}}}}{\sqrt{{{(c}_{eq})}^{\text{2}}+{\left(k_{eq}-\text{1}\right)}^{\text{2}}}} \end{array}$$ (33)

$$\begin{array}{c} \phi =\text{arctan}\left(\frac{c_{eq}}{k_{eq}-\text{1}}\right) \end{array}$$ (34)

#### 2.5.2. Analysis of axial vibration characteristics of metal braided hose.

The identified parameters [23] of the Bouc‑Wen model for the metal braided hose are listed in Table 1. The mass of the metal braided hose is 1.088 kg. The equivalent stiffness $k_{eq}$ and equivalent damping $c_{eq}$ are expressed by Eq. (21) and Eq. (28), respectively.

**Table 1:** Parameters of the axial Bouc-Wen model for metal braided hoses.

ρ	σ	u	$k_{E1}$	$k_{A0}$
1.5832	3.0416	1.3874	6.1730	40.26


$$\begin{array}{c} k_{eq}=\beta_{E\text{1}}=\frac{k_{Ei}}{m\omega^{\text{2}}}=\frac{\text{5}.\text{6}\text{7}\text{3}}{\omega^{\text{2}}} \end{array}$$ (35)

$$\begin{array}{c} c_{eq}=\frac{\beta_{A\text{0}}E}{\pi \lambda_{A}^{\text{2}}\omega }=\frac{k_{Aj}}{X\pi m{\lambda_{A}^{\text{2}}\omega }^{\text{3}}}E=\frac{\text{1}\text{1}.\text{7}\text{8}\text{5}}{{\lambda_{A}^{\text{2}}\omega }^{\text{3}}}E \end{array}$$ (36)

The dimensionless excitation force $\tilde{P_{\text{0}}}$ can be expressed as:


$$\begin{array}{c} \tilde{P_{\text{0}}}=\frac{P_{\text{0}}}{m\cdot X\cdot \omega^{\text{2}}}=\frac{P_{\text{0}}}{m\cdot \omega^{\text{2}}} \end{array}$$ (37)

Fig 4 shows the displacement response curves obtained using the above parameters. It can be seen from the Fig that the first two natural frequencies are 11.91 Hz and 19.60 Hz. When the excitation frequency exceeds 140 Hz, the displacement response of the metal braided hose undergoes significant deformation. To evaluate random uncertainties from experimental measurement noise and Bouc‑Wen parameter identification [28,29], residual‑bootstrap analysis is performed using near‑origin small‑displacement hysteresis data. The 95% confidence interval for the first‑order equivalent natural frequency of 11.9 Hz is [11.18, 12.66] Hz. Full parameter statistics and bootstrap frequency histogram are available in the S1 File.

**Fig 4:**
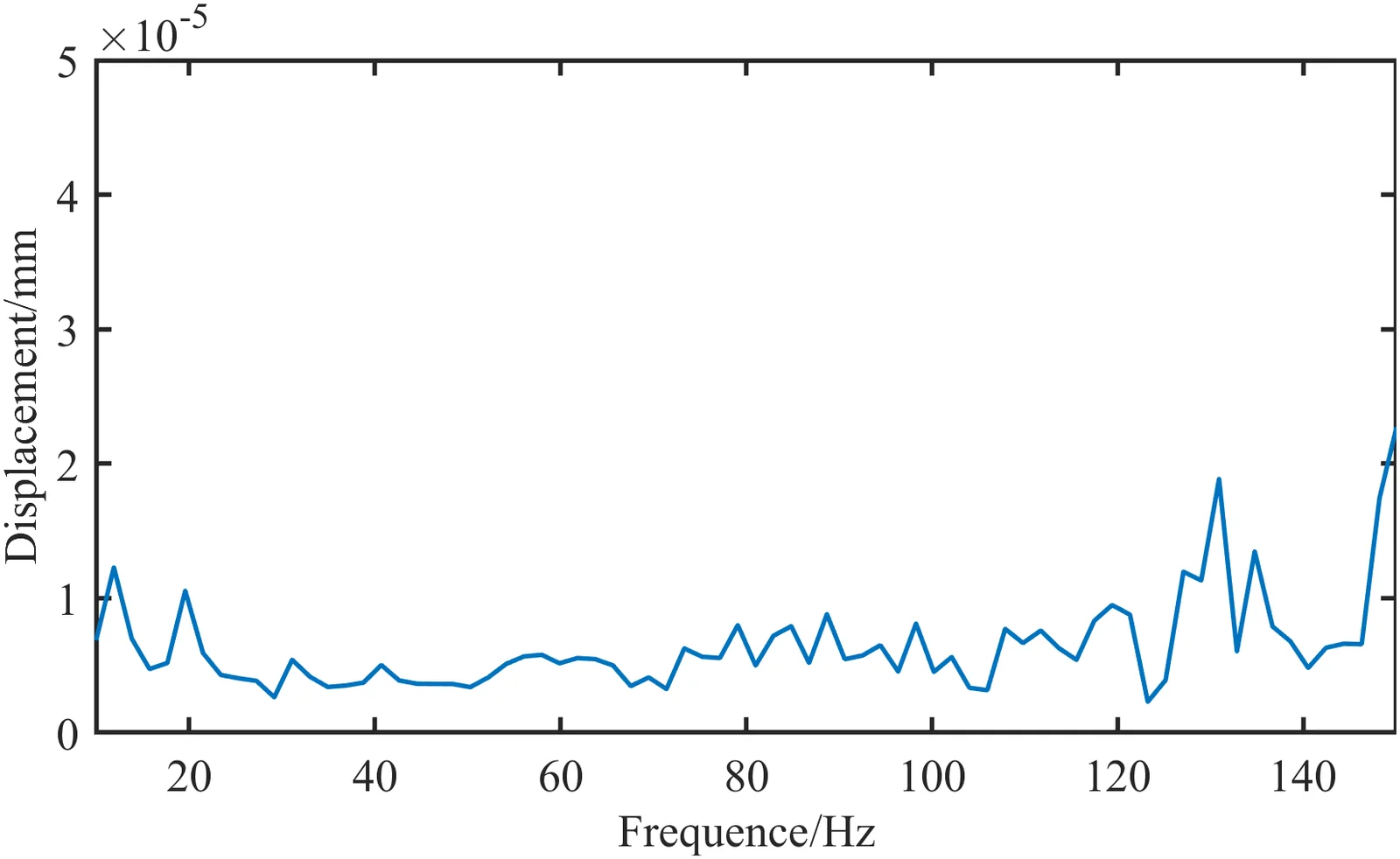
Amplitude-frequency response curve of axial vibration obtained from the equivalent linearized model.

#### 2.5.3. Analysis of bending vibration characteristics of metal braided hose.

The parameters of the Bouc-Wen model for the bending of the metal braided hose are shown in Table 2. The mass of the metal braided hose is 1.088 kg.

**Table 2:** Parameters of the bending Bouc-Wen model for metal braided hoses.

ρ	σ	u	$k_{E1}$	$k_{A0}$
1.6241	3.1247	1.4362	7.47	42.63

The displacement response curve of the metal braided hose is shown in Fig 5. It can be seen from the Fig that the first-order natural frequency is 13.2 Hz. To evaluate random uncertainties from experimental measurement noise and Bouc‑Wen parameter identification, residual‑bootstrap analysis is performed using near‑origin small‑displacement hysteresis data. The 95% confidence interval for the first‑order equivalent natural frequency of 13.2 Hz is [12.41, 14.12] Hz. Full parameter statistics and bootstrap frequency histogram are available in the S1 File.

**Fig 5:**
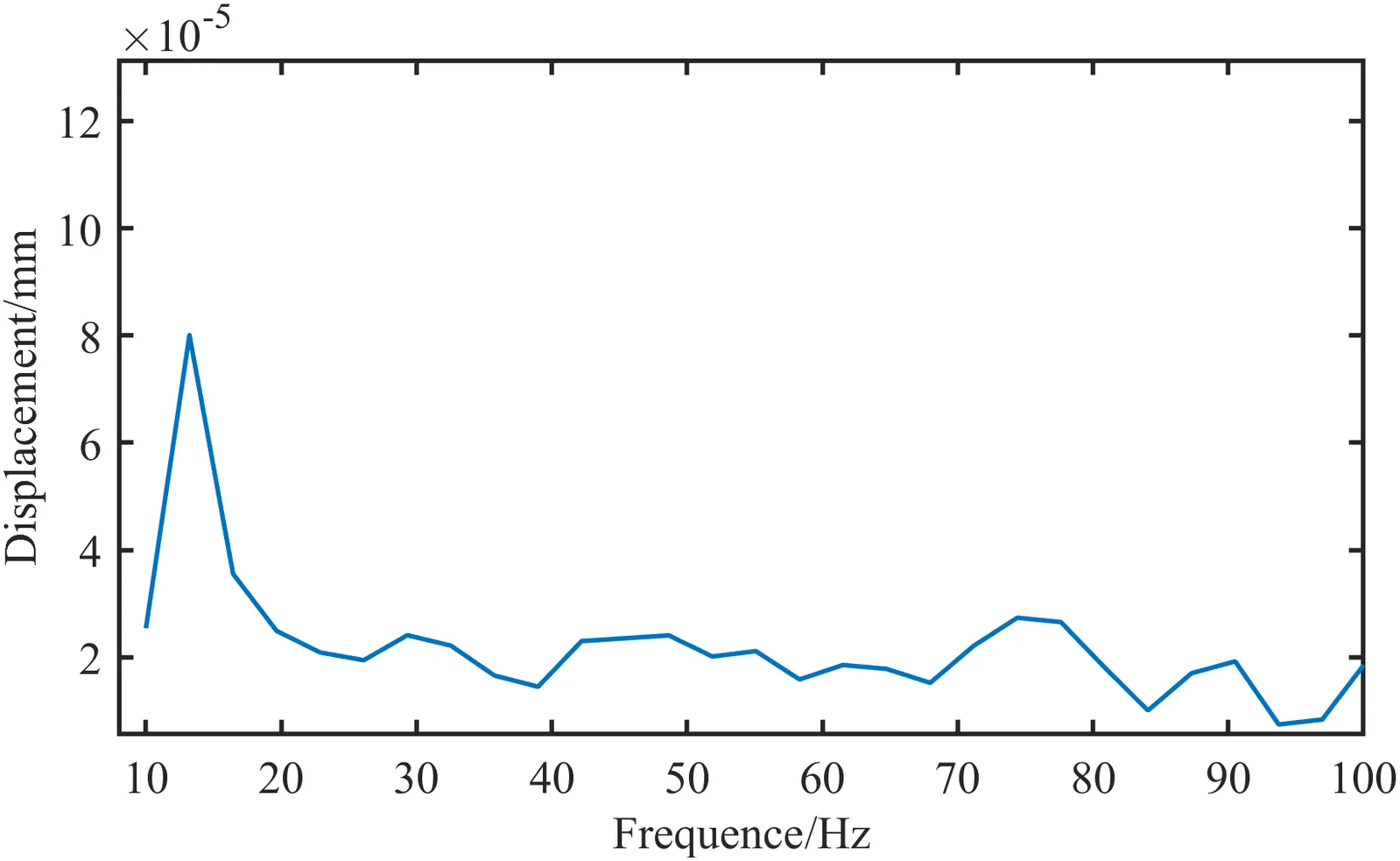
Amplitude-frequency response curve of bending vibration obtained from the equivalent linearized model.

## 3. Experiment

### 3.1. Experimental equipment

The experimental setup for the dynamic response of metal braided hoses consisted of a data acquisition system and a hardware system. The acquisition system was composed of a computer and an M + P data acquisition analyzer; the hardware system included a vibration exciter, a force sensor, acceleration sensors, and a displacement sensor. One end of the metal braided hose is fixed, and the other end is connected to the vibration exciter via a connecting rod. A force sensor was mounted on the connecting rod to monitor the excitation force, and a displacement sensor was installed at the joint to measure the axial displacement. Acceleration sensors were installed at both the excitation end and the constrained end, and the vibration isolation efficiency of the metal braided hose was evaluated by comparing the acceleration values.

Fig 6 (a) and (b) are schematic diagrams of the dynamic response of the metal braided hose under different excitation directions, respectively, and Fig 7 shows the experimental setup for axial excitation of the metal braided hose.

**Fig 6:**
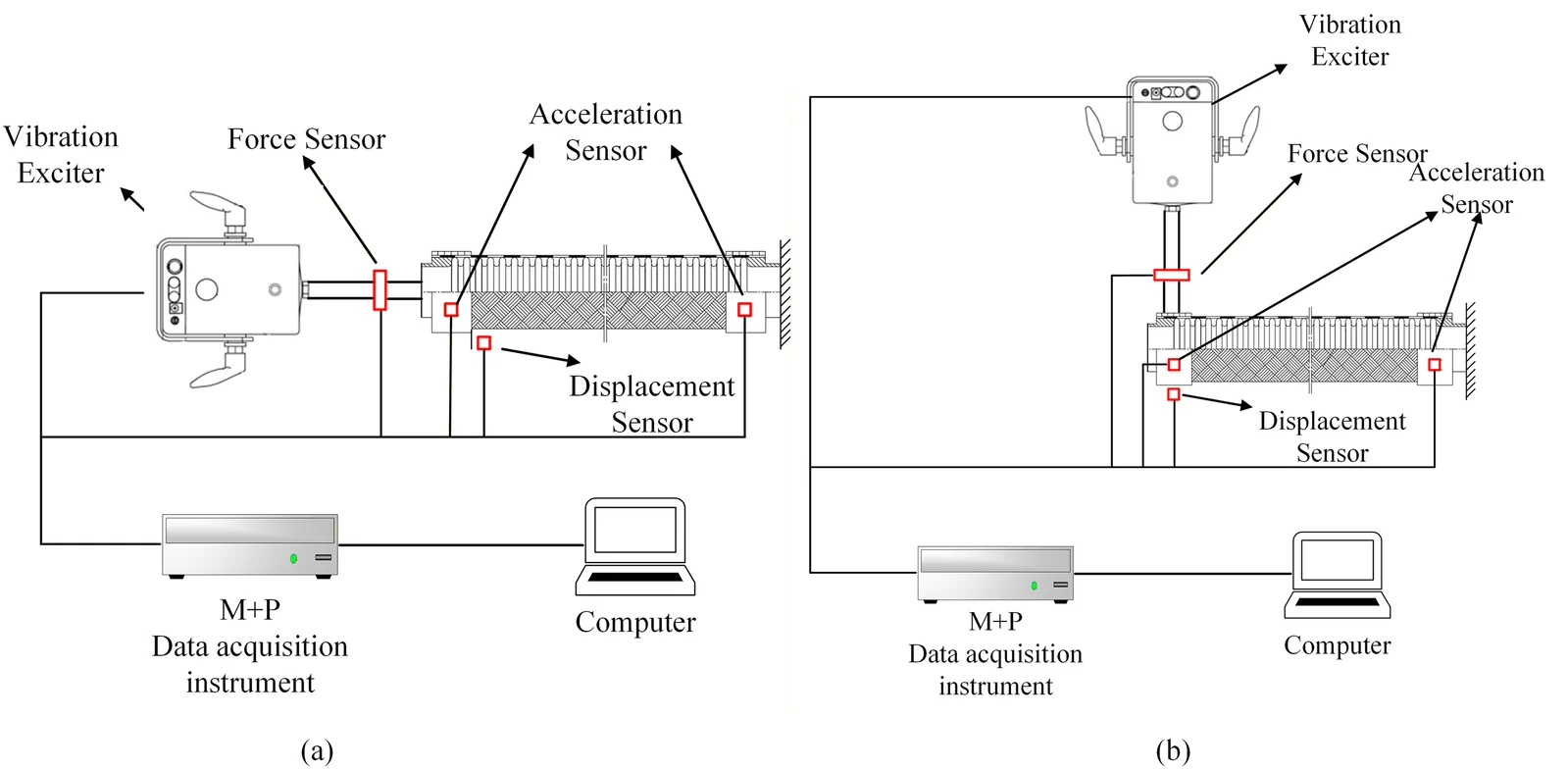
Schematic diagram of the dynamic response experiment of the metal braided hose (a) Axial dynamic response; (b) bending dynamic response.

**Fig 7:**
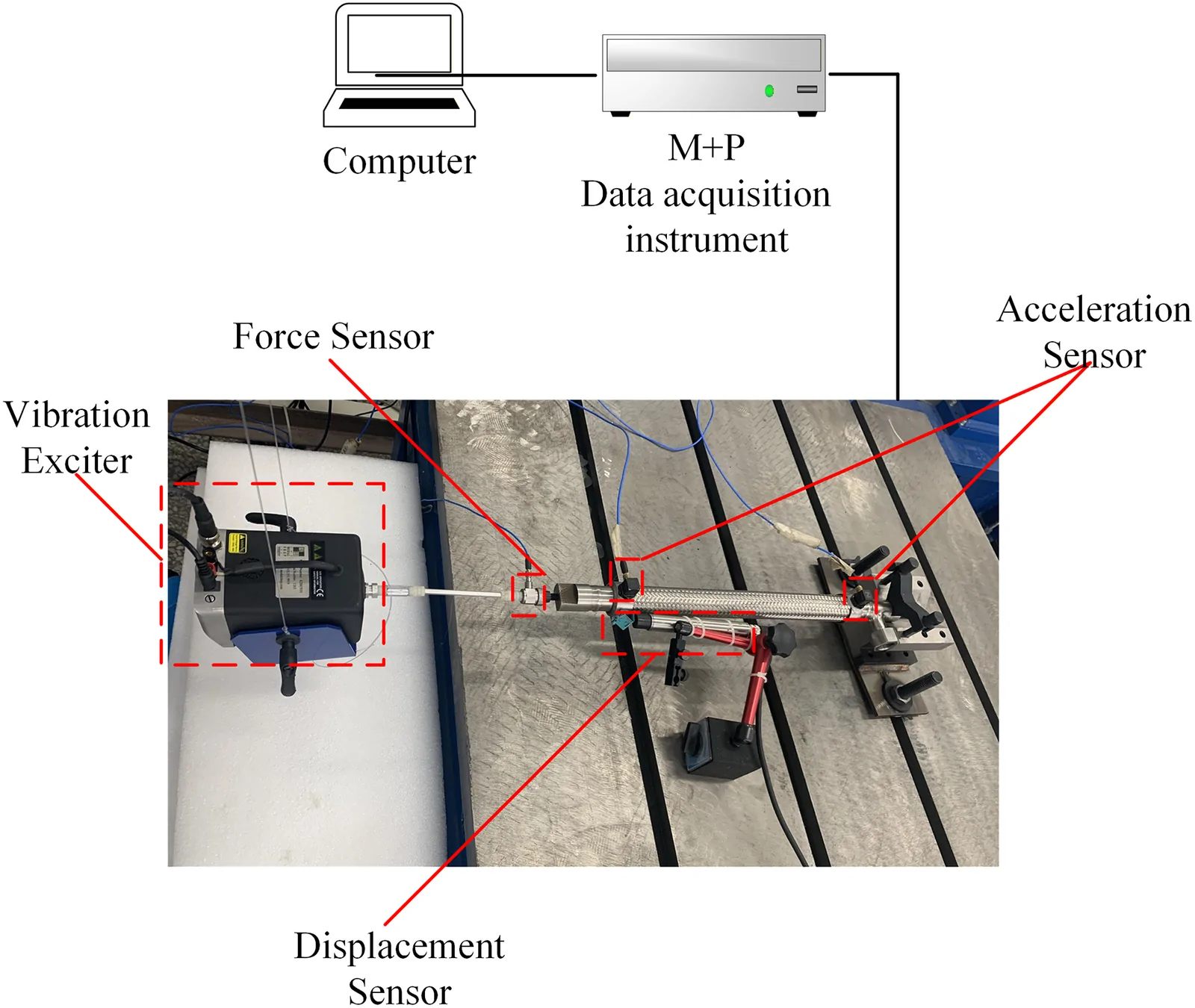
Diagram of the experimental device.

### 3.2. Experimental samples and setting conditions

The structural parameters of the corrugated tube used in the experiment and the structural parameters of the braided mesh sleeve are shown in Fig 8, Table 3, and Table 4. Five different types of metal braided hoses were used in this experiment, and the specimens are listed in Table 5.

**Table 3:** The structure of the bellows.

	L/mm	*D*/mm	$d_{in}$/mm	$r_{1}$/mm	$r_{2}$/mm	*t* /mm	$h_{0}$/mm	H/mm
DN25	222	31.5	25.4	0.8	0.8	3.7	0.25	2.8

**Table 4:** The structure of the braided mesh sleeve.

Strands	Number of pieces per strand	Braid angle	Diameter
36	12	45°	0.3mm

**Table 5:** Experimental sample.

Number	Diameter/mm	Length/mm	Number of layers of the braided mesh sleeve
1	DN25	220	1
2	DN25	220	2
3	DN25	220	3
4	DN25	320	1
5	DN50	220	1

**Fig 8:**
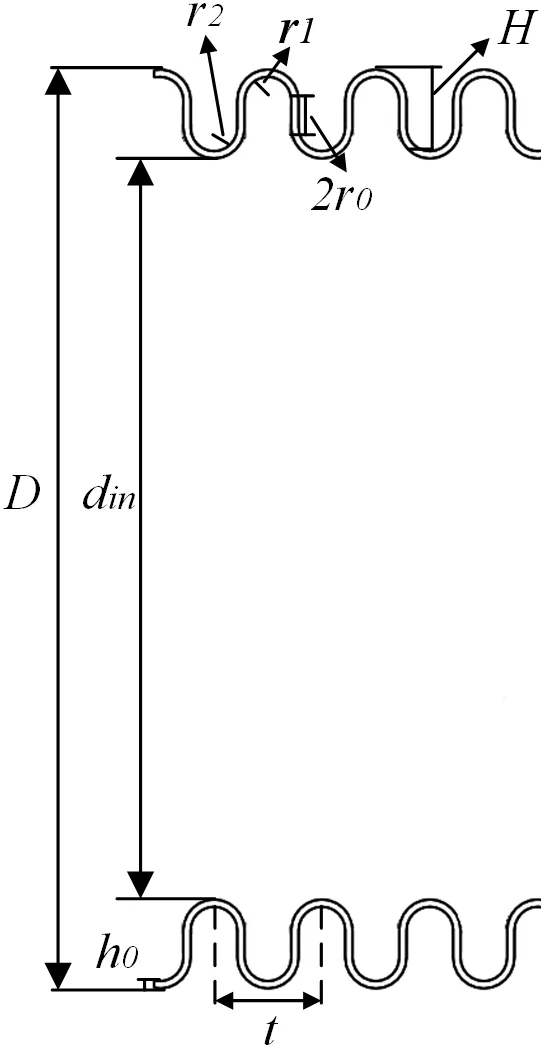
The structure of the bellows.

The vibration exciter used in the test can only be set with force amplitude and cannot be adjusted for displacement amplitude or acceleration amplitude. The excitation frequency in the experiment ranges from 5 Hz to 400 Hz, and the amplitude of the exciter was regulated by the voltage level. In this experiment, the exciter amplitude was set to 0.1 V.

## 4. Results

### 4.1. Analysis of axial vibration results

#### 4.1.1. Natural frequency of metal braided hose.

Fig 9 shows the displacement frequency response curves of different metal braided hoses. Generally, the displacement frequency response curve at low frequencies and the acceleration frequency response curve at high frequencies can better reflect the natural frequencies of the system. The excitation frequency in this experiment ranges from 5 Hz to 400 Hz. To clearly observe the low-frequency response, only the results from 5 Hz to 200 Hz were presented. The first-order natural frequencies of the five hoses were listed in Table 6.

**Table 6:** Natural frequency of the metal braided hose.

Hose Number	Natural frequency / Hz	
	First-order	Second-order
1	10	18
2	9	22
3	10	18
4	10	16
5	11	22

**Fig 9:**
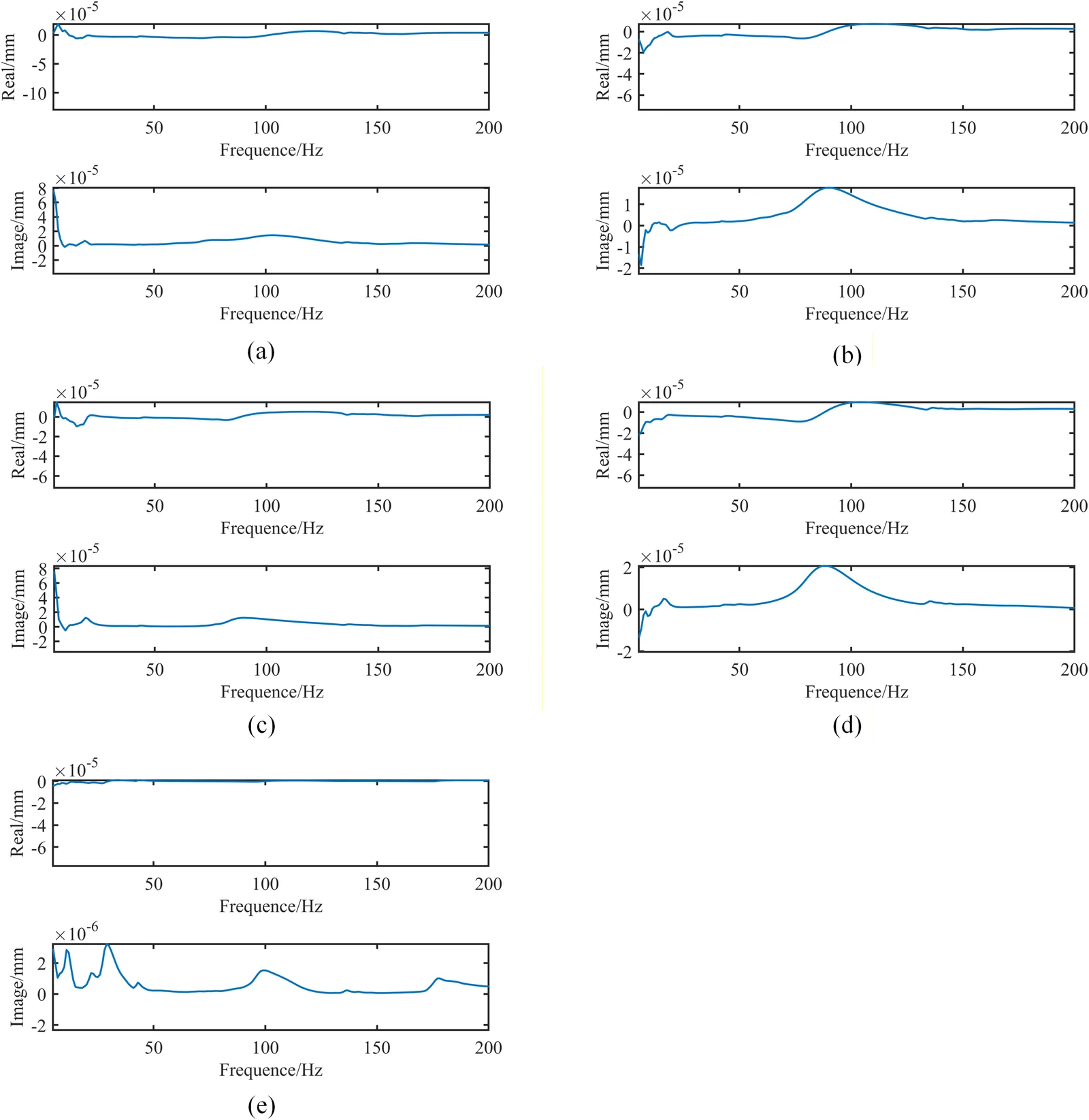
Displacement frequency response curve of the metal braided hose. (a) No.1; (b) No.2; (c) No.3; (d) No.4; (e) No.5.

It can be seen from Table 6 that the first‑order natural frequencies of the five metal braided hoses are approximately 10 Hz, and the second‑order natural frequencies range from 16 Hz to 22 Hz. Hose No. 4 has the lowest second‑order natural frequency due to its longer length and lower stiffness.

#### 4.1.2. Amplitude-frequency response analysis of metal braided hose.

Fig 10 shows the amplitude response curves of different metal braided hoses within 5–400 Hz. Despite differences in structure and diameter, the responses of all hoses are concentrated within 150 Hz, indicating that their axial vibration is prone to low-frequency resonance. The displacement response is significantly enhanced near 10–20 Hz and 80 Hz, corresponding to the natural frequency ranges, which is basically consistent with the results of the Frequency Response Function (FRF).

**Fig 10:**
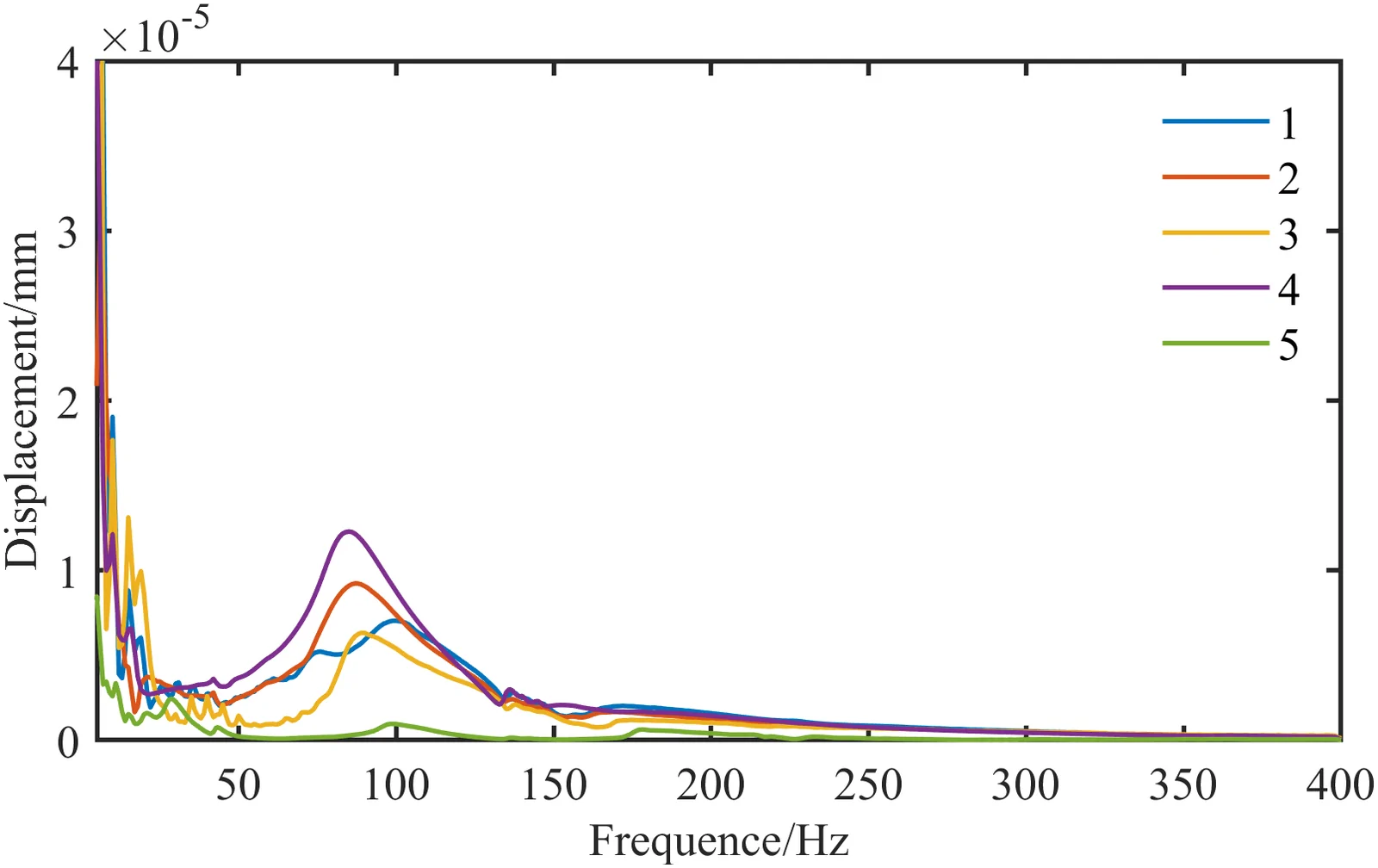
Amplitude-frequency curve of axial vibration.

Fig 11 shows the comparison of the amplitude-frequency curves of the experiments and numerical calculations within 8–100 Hz. The natural frequencies from both approaches are relatively close. However, since equivalent stiffness and equivalent damping are adopted in the numerical model, the calculated amplitude in the first-order resonance region is smaller than the experimental value.

**Fig 11:**
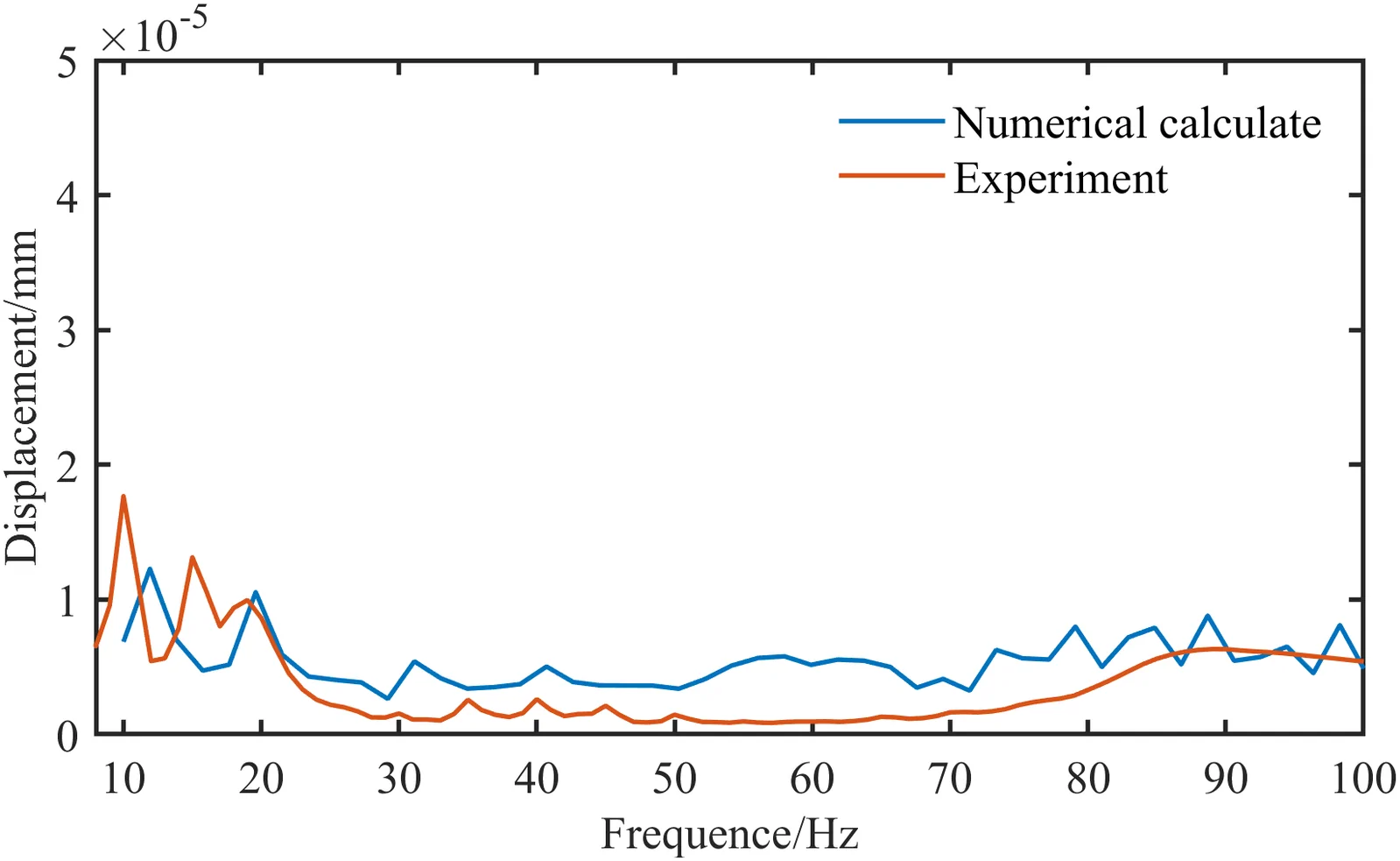
Comparison of the axial vibration response curves between experiments and numerical calculations within 8-100 Hz.

#### 4.1.3. Vibration isolation performance of metal braided hose.

In this paper, the acceleration vibration level difference is used to evaluate the vibration isolation efficiency of the metal braided hose. The formula for the acceleration vibration level difference is:


$$\begin{array}{c} L_{D}=\text{2}\text{0}lg\left(\frac{a\text{1}}{a\text{2}}\right) \end{array}$$ (38)

where a1 and a2 are the effective acceleration values at the input and output ends, respectively.

Fig 12 shows the time-domain acceleration signals at the input and output ends of the metal braided hose under axial vibration, which indicates a good vibration isolation effect. The overall vibration isolation rate can be obtained by comparing the effective acceleration values within 5–400 Hz. The effective acceleration values of the five metal braided hoses are listed in Table 7.

**Table 7:** Effective acceleration and vibration level difference at the input and output ends under axial vibration of the metal braided hose.

No	Effective input acceleration / $m/s^{2}$	Effective output acceleration / $m/s^{2}$	Vibration level difference / dB
1	20.551	5.0038	12.27
2	20.575	4.6938	12.83
3	17.072	4.4915	11.60
4	23.252	5.61	12.35
5	3.83	1.56	7.27

**Fig 12:**
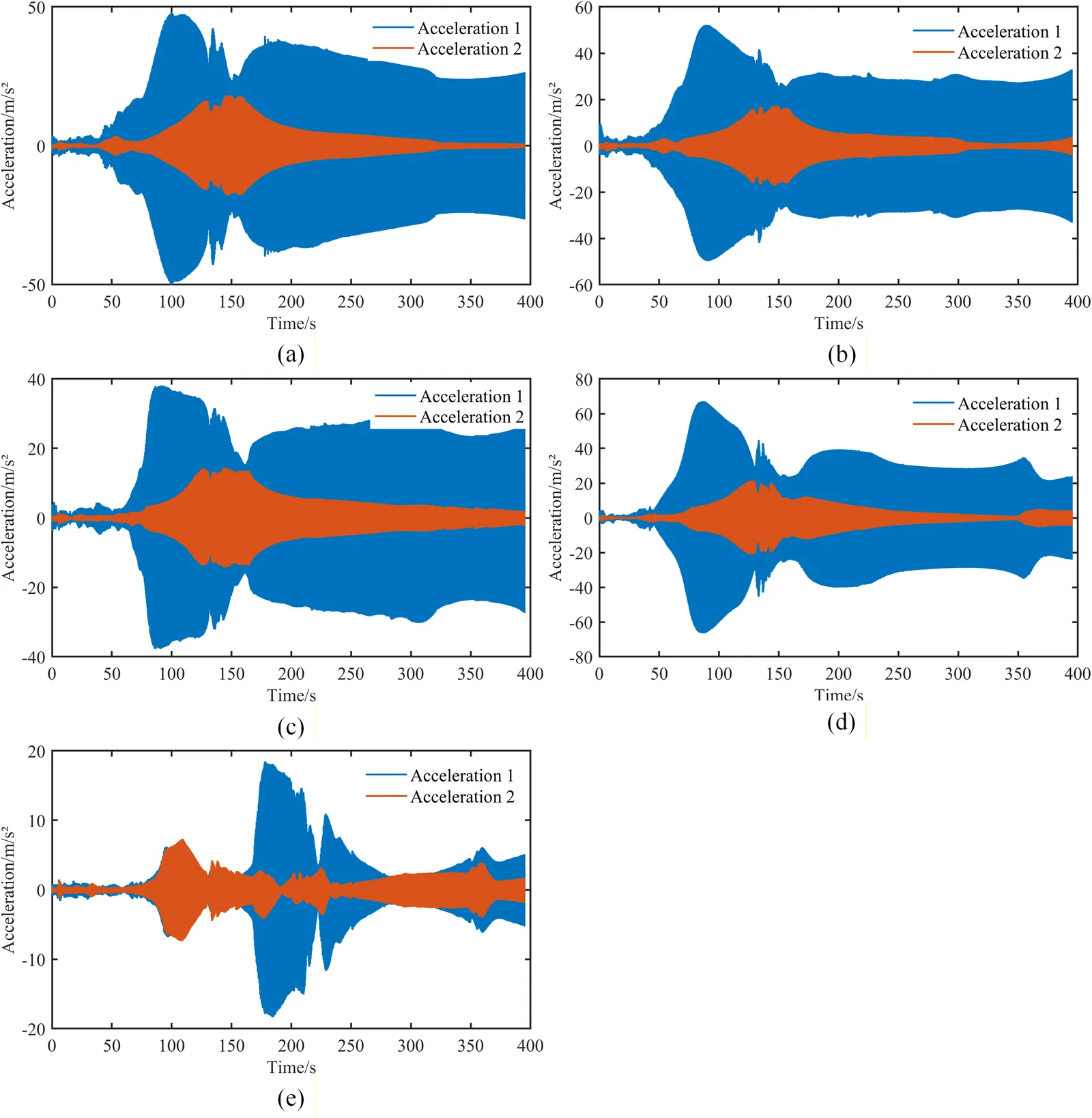
Time-domain acceleration signals at the input and output ends under axial vibration of the metal braided hose. (a) No.1; (b) No.2; (c) No.3; (d) No.4; (e) No.5.

As shown in Table 7, the vibration isolation effect of DN25 is better than that of DN50, because DN50 has a larger diameter and higher axial stiffness, resulting in weaker vibration attenuation. Among DN25 hoses, the two-layer braided hose exhibited the highest vibration isolation rate, which was 12.83 dB, while the three-layer one showed the lowest, which was 11.60 dB. Although increasing the number of braided layers improved damping, it also increased axial stiffness, so the overall improvement in vibration isolation was not significant. Hose No. 1 and No. 4, which differ in length, had similar vibration isolation rates. Due to the bending deformation generated in the long hose under excitation, the bending stiffness jointly affected the axial response, making its axial vibration isolation effect slightly inferior to that of the short hose.

Fig 13 shows the acceleration spectra and vibration level differences of different metal braided hoses. DN25 exhibits excellent overall vibration isolation performance. In the initial frequency band, the vibration level difference was close to 0 dB due to natural resonance and gradually increased with rising frequency. It decreased slightly to nearly 150 Hz owing to the resonance at the output, and the vibration isolation effect remained stable within 400 Hz. DN50 only provided favorable vibration isolation in the ranges of 0–50 Hz, 160–280 Hz, and 320–400 Hz, while vibration amplification occurred in other frequency bands. Therefore, large-diameter metal braided hoses, characterized by high axial stiffness and insufficient braided damping, require additional damping layers to improve their vibration isolation performance.

**Fig 13:**
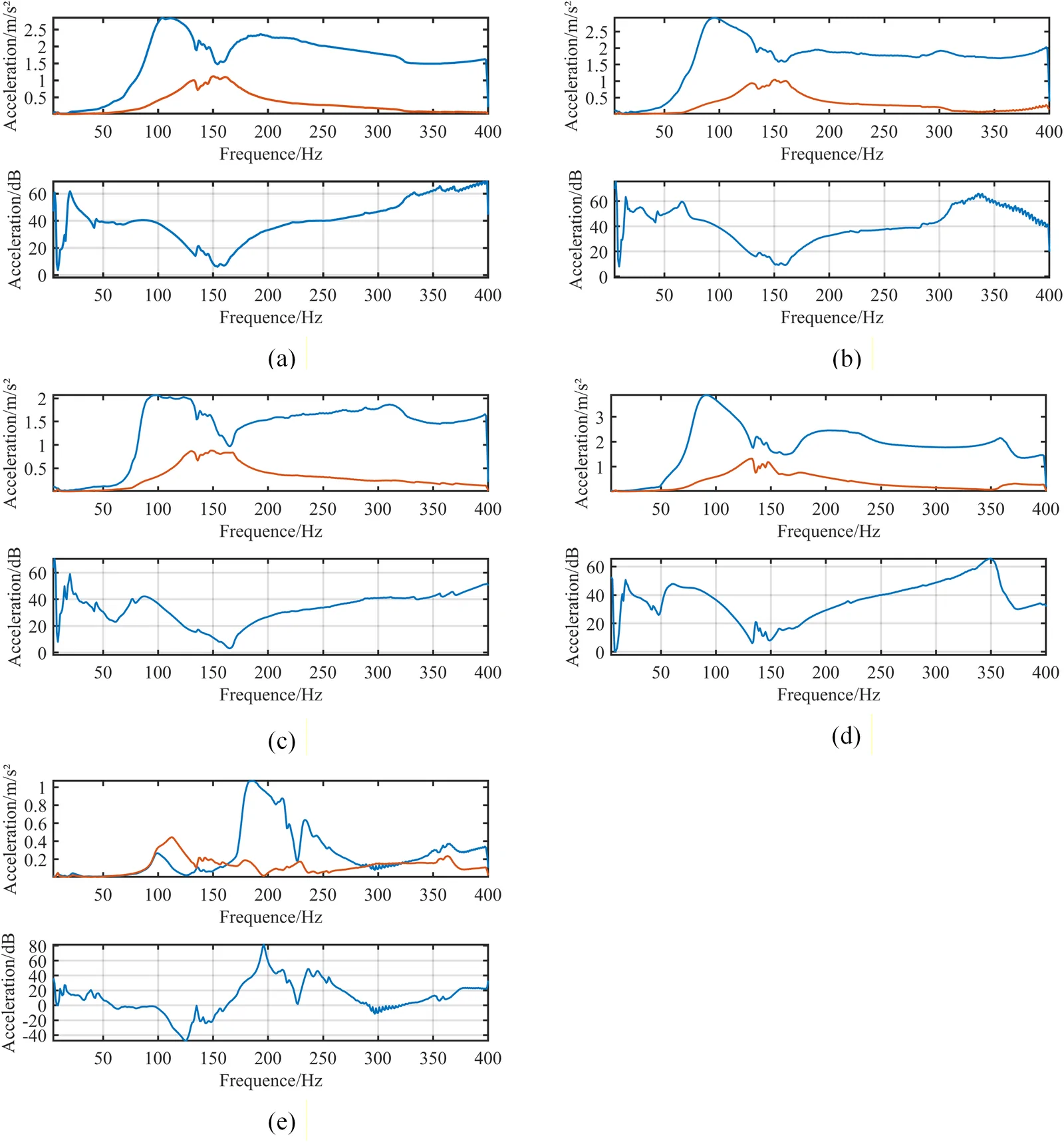
Vibration level difference of axial vibration acceleration signals for metal braided hoses. (a) No.1; (b) No.2; (c) No.3; (d) No.4; (e) No.5.

### 4.2. Analysis of bending vibration results

#### 4.2.1. Natural frequency of metal braided hose.

Fig 14 shows the displacement frequency response curves of different metal braided hoses, whose first‑order natural frequencies are listed in Table 8. The long DN25 hose had a natural frequency of 8 Hz due to its greater length and lower stiffness. The DN50 hose had a slightly higher natural frequency of 14 Hz, while the other DN25 hoses with different braiding layers all had a natural frequency of 12 Hz.

**Table 8:** Natural frequency of the metal braided hose.

Number	Natural frequency / Hz
	First-order
1	12
2	12
3	12
4	8
5	14

**Fig 14:**
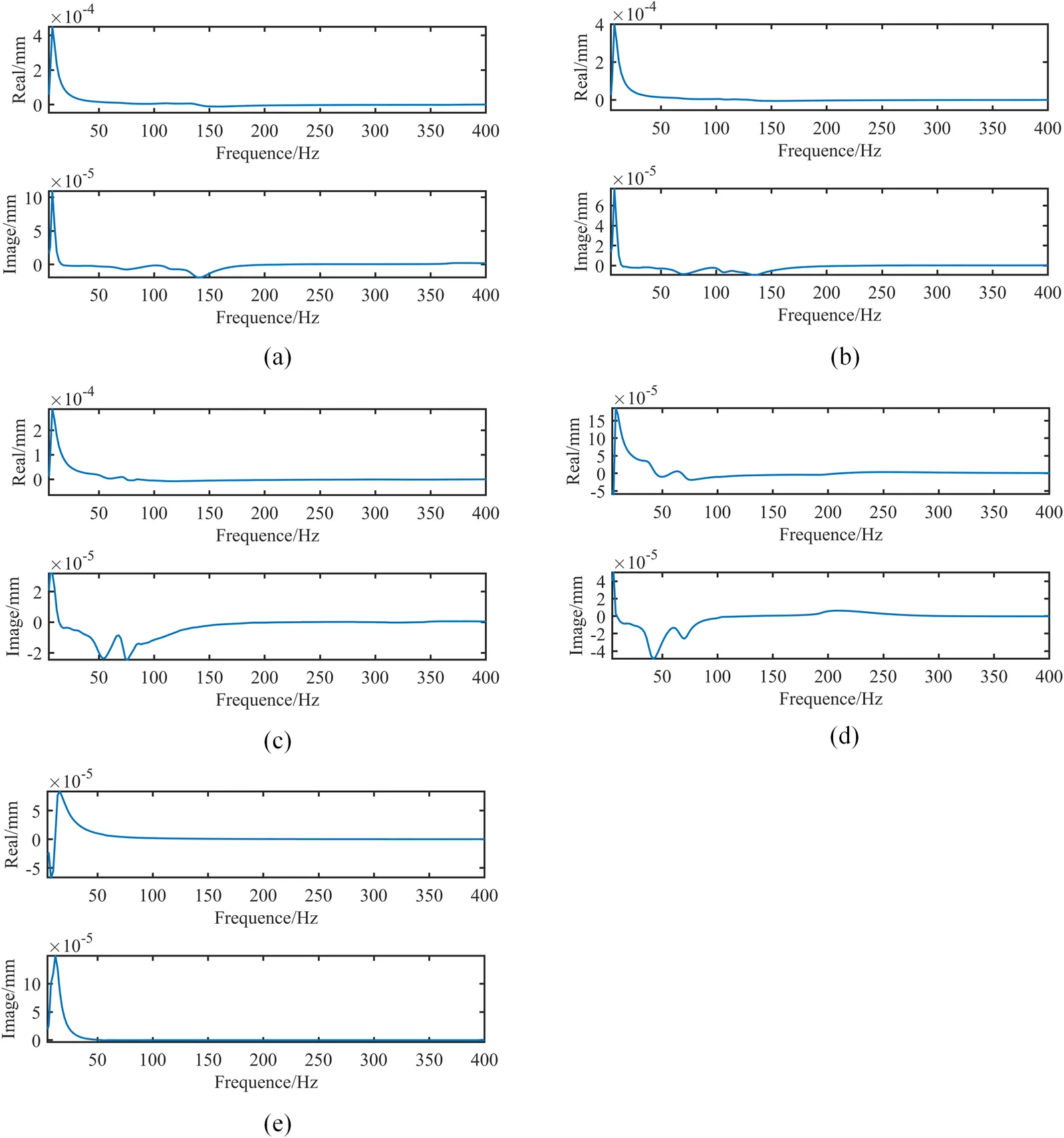
Displacement frequency response curve of the metal braided hose. (a) No.1; (b) No.2; (c) No.3; (d) No.4; (e) No.5.

#### 4.2.2. Amplitude-frequency response analysis of metal braided hose.

Fig 15 shows the amplitude response curves of different metal braided hoses within 5–400 Hz. Despite differences in structure and diameter, the large-amplitude responses of all hoses were concentrated within 150 Hz, indicating that their bending vibration was prone to low-frequency resonance. The displacement response was significantly higher near 8–20 Hz and 40–80 Hz, corresponding to the natural frequency ranges, which were basically consistent with the results of the frequency response function.

**Fig 15:**
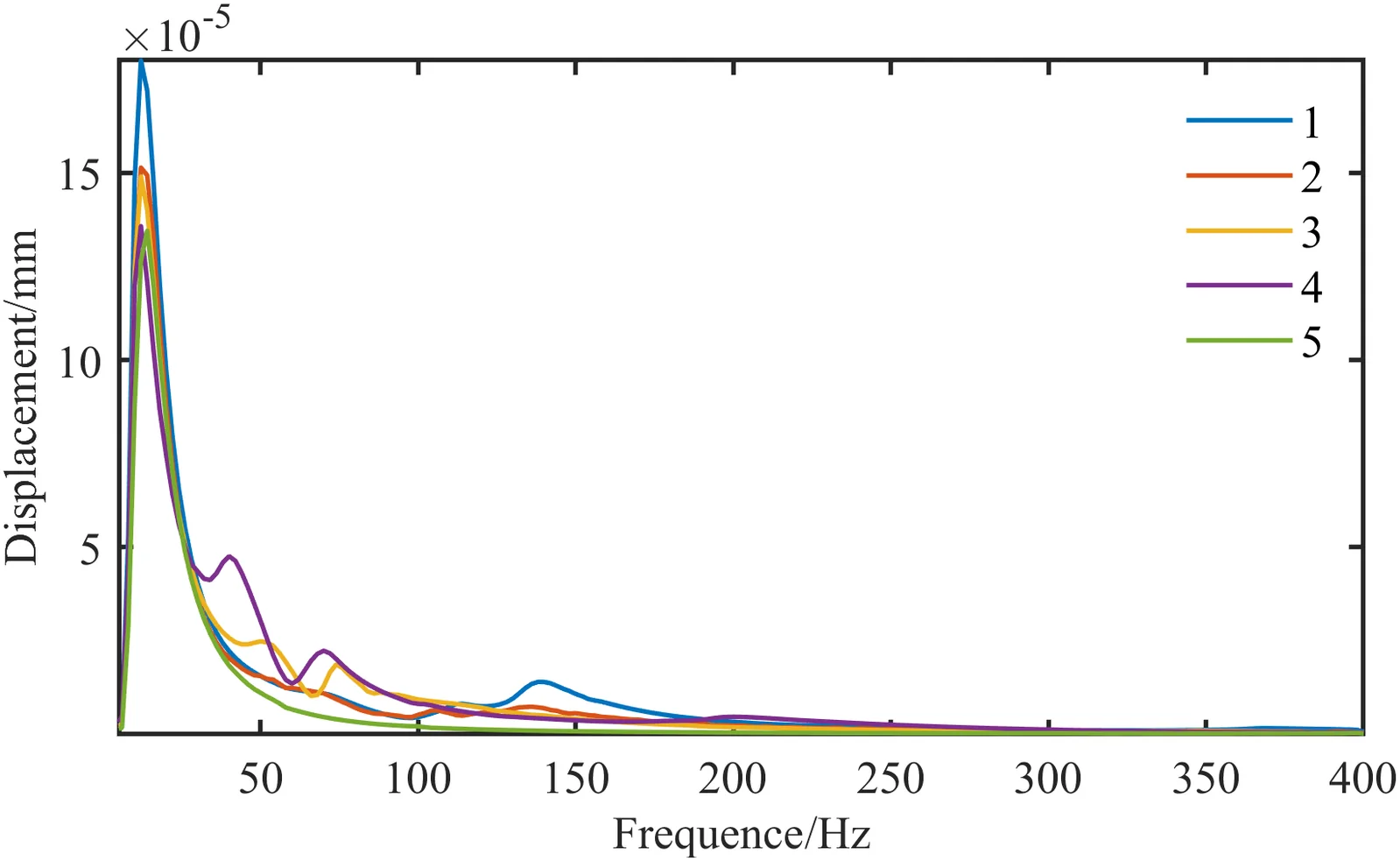
Amplitude-frequency curve of bending vibration.

Fig 16 compares the amplitude-frequency curves obtained from experiments and numerical calculations within 8–100 Hz, and the natural frequencies of the two were close. Since equivalent stiffness and equivalent damping were adopted in the numerical calculation, the amplitude in the first-order resonance region was slightly different from the experimental value.

**Fig 16:**
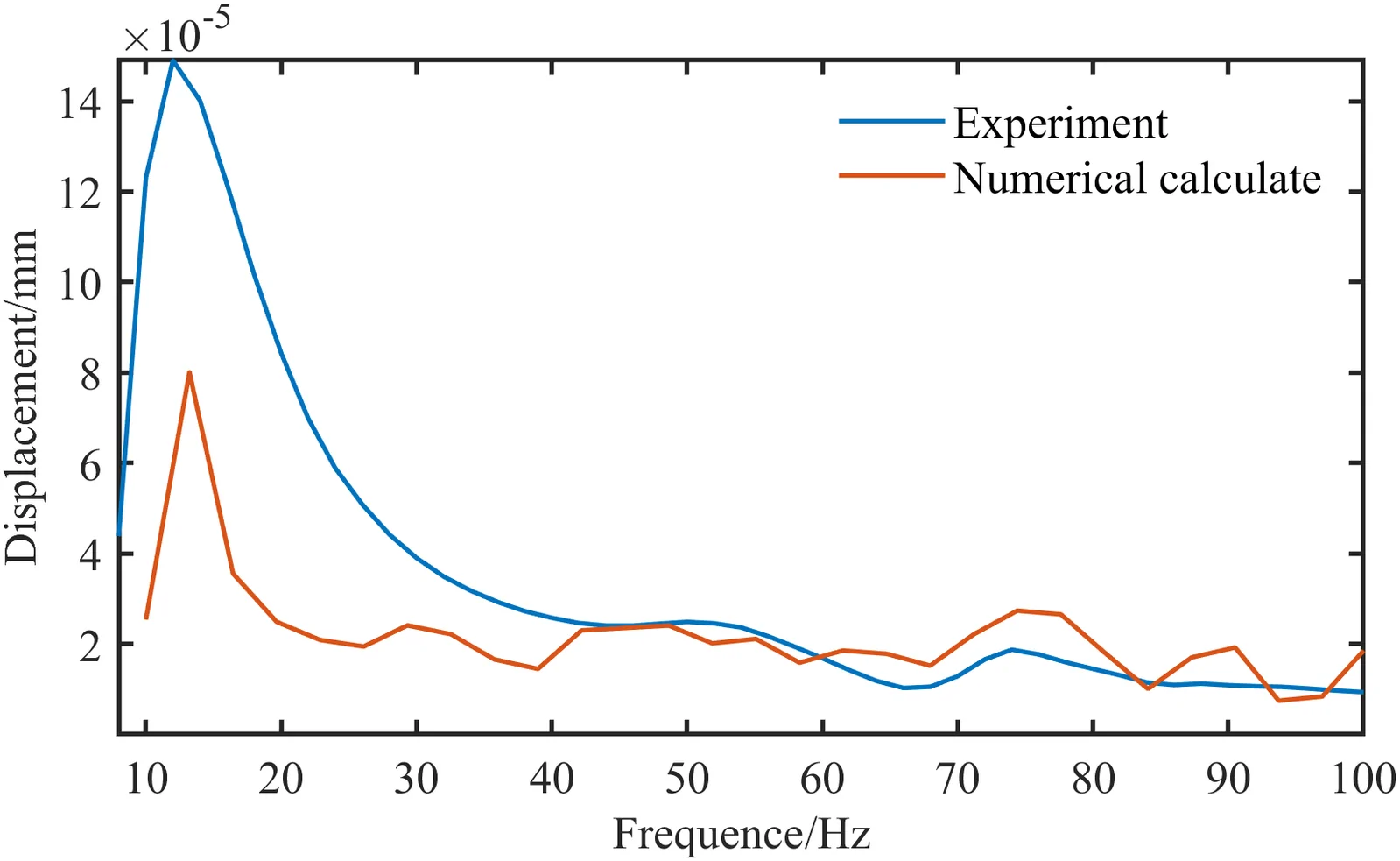
Comparison of the bending vibration response curves between experiments and numerical calculations within 8-100 Hz.

#### 4.2.3. Vibration isolation performance of metal braided hose.

Fig 17 shows the time-domain acceleration signals at the input and output ends of the metal braided hose under bending vibration, indicating a favorable vibration isolation effect. The overall vibration isolation rate can be obtained by comparing the effective acceleration values within 5–400 Hz. The effective acceleration values of the five metal braided hoses are presented in Table 9.

**Table 9:** The effective value of the acceleration and vibration level difference at the input and output ends of the metal braided hose, due to bending vibration.

Number	Input acceleration effective value / $m/s^{2}$	Output acceleration effective value / $m/s^{2}$	Vibration level difference / dB
1	21.562	1.6204	22.481
2	18.676	1.2371	23.578
3	17.713	1.2245	23.207
4	18.36	0.9783	25.46
5	8.9736	6.6685	2.579

**Fig 17:**
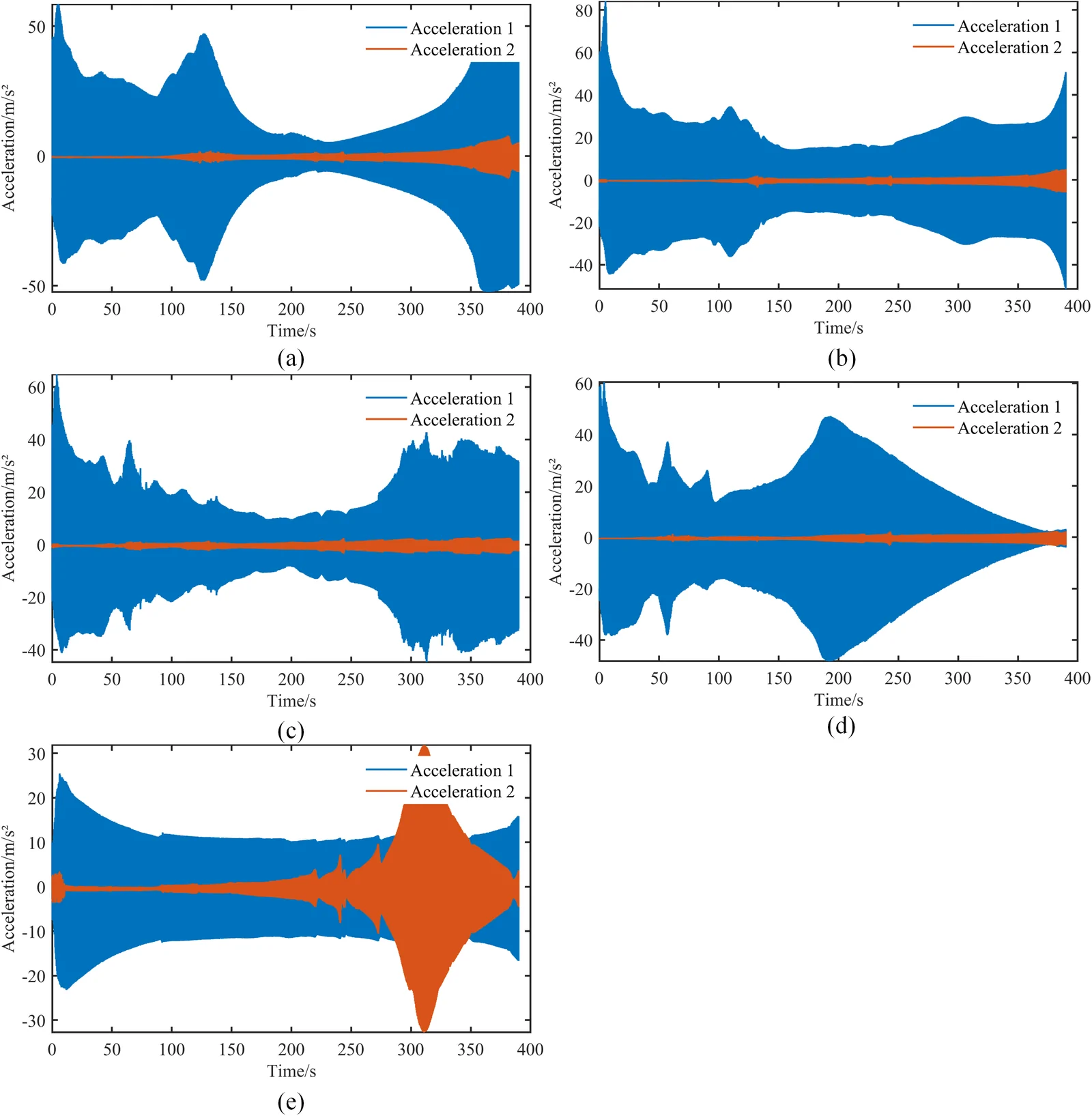
Time-domain acceleration signals at the input and output ends of metal braided hoses under bending vibration. (a) No.1; (b) No.2; (c) No.3; (d) No.4; (e) No.5.

As shown in Table 9, the vibration isolation effect of DN25 is better than that of DN50, since DN50 has a higher bending stiffness and weaker vibration attenuation. DN25 hoses exhibited excellent overall vibration isolation performance, among which the long single-layer braided hose performed the best, followed by the short single-layer braided hose. Although increasing the number of braided layers improved damping, it also increased bending stiffness, resulting in the largest vibration level difference for the two-layer braided hose. The vibration level difference between Hose No. 1 and No. 4 differed by 3 dB, mainly because the increase in length reduced axial stiffness and increased damping, leading to better vibration isolation performance.

Fig 18 shows the acceleration spectra and vibration level differences of various metal braided hoses. DN25 demonstrated favorable overall vibration isolation performance. Hoses 1, 2, and 3 exhibited excellent vibration isolation, with vibration level differences exceeding 50 dB across the entire frequency range. The vibration level difference of Hose 4 dropped sharply at 390 Hz, yet overall vibration isolation was still achieved. Hose 5 showed relatively poor vibration isolation between 300 Hz and 350 Hz, and time-domain results indicated an increase in amplitude within this frequency band, which should be avoided in application.

**Fig 18:**
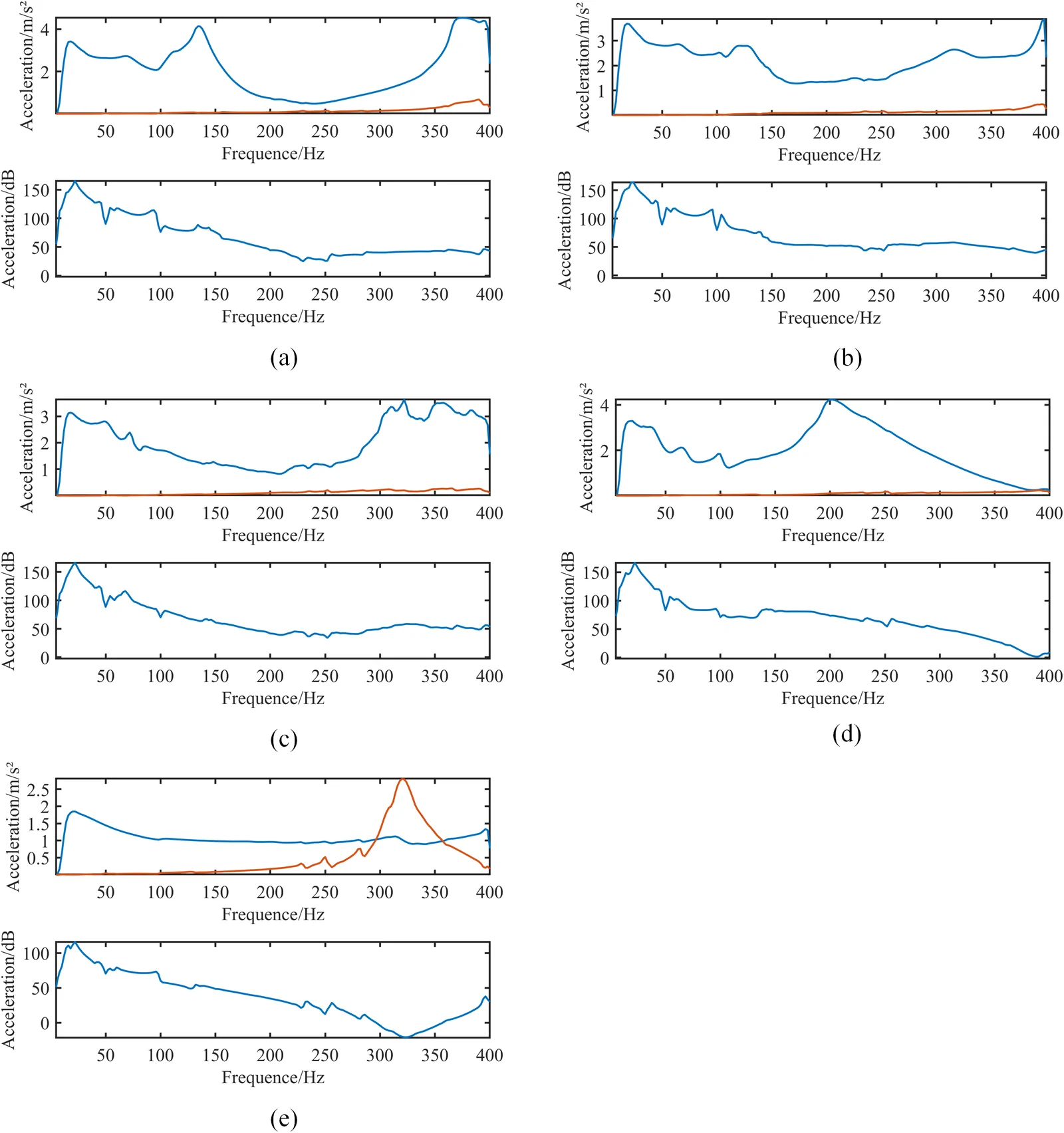
The amplitude difference of the vibration acceleration signal of the metal braided hose bending. (a) No.1; (b) No.2; (c) No.3; (d) No.4; (e) No.5.

By comparing the axial and bending vibration level differences of metal braided hoses, the bending vibration isolation effect was superior. Under the same braiding parameters, the bending stiffness of the hose was lower than its axial stiffness, resulting in better vibration isolation performance in bending than in the axial direction.

## 5. Discussion

This study investigates axial and bending hysteresis behaviours of metal braided hoses using an improved normalized Bouc‑Wen model combined with quasi‑static and dynamic tests. Large‑deformation stiffness‑hardening is clearly observed, and the proposed model satisfactorily reproduces both asymmetric axial and symmetric bending hysteresis loops. Equivalent natural frequency and vibration‑isolation performance are further derived via energy‑based equivalent linearization for small‑amplitude low‑frequency conditions.

Compared with existing hose‑dynamic studies relying on classical Bouc‑Wen formulations, the modified hysteresis evolution equation in this work better captures stiffness‑hardening and asymmetric hysteresis characteristics of braided‑mesh hoses. As a semi‑empirical modelling approach, it offers an efficient alternative to computationally heavy finite‑element contact simulations and facilitates system‑level pipeline vibration analysis.

Physically, stiffness‑hardening arises from progressive contact and squeezing between the braided mesh and corrugated bellows with growing displacement. At infinitesimal displacement, the mesh remains non‑contact and yields low initial tangent stiffness. Hysteretic energy dissipation is dominated by interfacial friction among braided metal wires, leading to amplitude‑dependent stiffness and damping that govern the hose’s dynamic performance.

Several limitations and multi‑source uncertainties should be acknowledged. Stochastic uncertainty from experimental noise and parameter identification is quantified via residual‑bootstrap. Key statistics including 95% confidence intervals of equivalent natural frequencies and sensitivity results are provided in the S2 File. Sensitivity analysis reveals that small‑displacement natural frequency is mainly controlled by elastic stiffness $k_{E1}$, while hysteresis parameters predominantly affect equivalent damping and force‑transmission ratio. The energy‑equivalence equivalent‑linearization is strictly valid only for small‑amplitude low‑frequency excitation and cannot be extrapolated to large‑amplitude or high‑frequency regimes. Limited tested specimens introduce generalizability constraints; measurement uncertainty for vibration indexes is also given in the S3 File. Besides these lab‑originated uncertainties, real‑world engineering factors including random loads, manufacturing scatter, material degradation, and variable service environments are not incorporated in the present model, representing epistemic uncertainty.

From an engineering viewpoint, the present modelling workflow supports vibration‑isolation assessment of hose‑equipped pipelines under small‑amplitude low‑frequency conditions. Further reliability‑oriented uncertainty‑aware design considerations are suggested for practical industrial applications, as outlined in the concluding outlook.

## 6. Conclusion and future work

This study investigated the dynamic response characteristics and vibration isolation mechanism of metal braided hoses. A dynamic equation was constructed based on the Bouc-Wen model, incorporating the stiffness and damping hysteresis properties of metal braided hoses, which was further simplified via the equivalent linearization method for the numerical solution of axial and bending displacement responses. Numerical results were compared with experimental data, validating the accuracy and reliability of the adopted method within a specific frequency range. Dynamic tests in both axial and bending directions were conducted on five metal braided hose specimens. The results revealed that all specimens possessed close natural frequencies, attributed to the small mass proportion of bellows and braided mesh sleeves. Although the numerically predicted first-order and first two natural frequencies agreed well with the experimental measurements, differences in vibration amplitudes remained due to numerical equivalent simplification errors. Vibration isolation performance analysis demonstrated that pipe diameter served as the key influencing factor; DN50 hoses showed inferior vibration level difference and isolation performance compared with DN25 hoses because of their higher axial and bending stiffness. For DN25 hoses, vibration isolation performance was affected by braided layer number and hose length: more braided layers simultaneously increased damping and bending stiffness; the three-layer braided hose had slightly poorer axial isolation, the two-layer braided hose presented the largest vibration level difference, and the longer DN25 hose exhibited the optimal vibration isolation effect. This work provides a basis for subsequent engineering‑oriented research. Future studies may go beyond deterministic dynamic analysis for reliability‑oriented and uncertainty‑aware design: carrying out probabilistic parameter identification using the residual‑bootstrap samples obtained in this work; performing durability and fatigue‑damage analysis based on hysteresis energy from the improved Bouc‑Wen model; implementing structural optimization under uncertain operating conditions considering load fluctuations and manufacturing scatter, so as to further improve the engineering applicability of metal‑braided hose models.

## Supporting information

S1 File Residual‑Bootstrap uncertainty quantification method. (DOCX)

S2 File Sensitivity analysis for model parameters and equivalent damping assumption. (DOCX)

S3 File Measurement uncertainty for measured vibration‑isolation quantities. (DOCX)
