# Supplementary material for: Equivalent linearization modeling and experimental validation of natural frequency and vibration isolation performance in metal braided hoses
Source: PLoS One. 2026 Nov 3;21(11):e0361869. doi: 10.1371/journal.pone.0361869 (PMC13730378; doi:10.1371/journal.pone.0361869)
Supplement: Supplementary file 1 [file pone.0361869.s001.docx]

**S1 Residual‑Bootstrap uncertainty quantification method**

To evaluate the stochastic uncertainty originating from restoring‑force sensor measurement noise and the ill‑posed parameter identification of the improved Bouc‑Wen model, residual‑bootstrap is adopted in this work.

The hysteresis test is a displacement‑controlled quasi‑static test. The displacement signal is closed‑loop controlled by the testing machine, so the uncertainty mainly comes from the restoring‑force measurement channel.

The reported first‑order equivalent natural frequency corresponds to the small‑displacement state before the contact between braided mesh and bellows. Full large‑amplitude axial hysteresis loops contain significant stiffness‑hardening effect induced by mesh‑bellows contact. If the full large‑amplitude hysteresis data are used for identification, the optimizer will be dominated by large‑force, large‑displacement points, and the identified stiffness will be the hardened large‑deformation stiffness rather than the initial stiffness for axial natural‑frequency calculation. Therefore, only the near‑origin small‑displacement segments$\left| x \right|\leq0.5 \mathrm{mm}$ extracted from multiple groups of axial hysteresis loops are used for uncertainty analysis.

A total of 200 bootstrap trials were carried out. Diverged and abnormal samples are discarded. The mean value, standard deviation, and 95% percentile confidence intervals of model parameters and axial natural frequency are statistically calculated.

**S1.1 Axial vibration**

Statistical results obtained from residual‑bootstrap based on the axial small‑displacement dataset. The 95% confidence intervals are calculated by the percentile method. Number of valid samples after removing diverged identification cases: 176.

**S1 Table Statistical results of improved axial Bouc‑Wen parameters and first‑order axial equivalent natural frequency from residual‑bootstrap**

| **parameter** | **symbol** | **Original identification value** | **bootstrap means** | **standard deviation** | **Lower 95% confidence limit** | **Upper 95% confidence limit** |
| --- | --- | --- | --- | --- | --- | --- |
| Hysteresis evolution coefficient | $\rho$ | 1.5832 | 1.5946 | 0.1742 | 1.2413 | 1.9126 |
| Hysteresis shape coefficient | $\sigma$ | 3.0416 | 3.0124 | 0.2615 | 2.4847 | 3.5218 |
| hardening exponent | u | 1.3874 | 1.3791 | 0.1086 | 1.1622 | 1.5843 |
| Axial tangential stiffness of small displacement | $k_{E1}/\left( N\cdot mm^{-1} \right)$ | 6.1730 | 6.1942 | 0.3826 | 5.5941 | 6.7824 |
| Hysteresis amplification coefficient | $k_{A0}/N$ | 40.2614 | 40.7135 | 3.4152 | 33.6271 | 47.1846 |
| Axial first-order equivalent natural frequency | $f_{n}/\mathrm{Hz}$ | 11.91 | 11.92 | 0.37 | 11.18 | 12.66 |

Histogram of axial first‑order equivalent natural frequency obtained from bootstrap sampling. The red vertical line represents the original identified axial natural frequency $f_{n}=11.91 \mathrm{Hz}$. Two green dashed lines denote the bounds of 95% confidence interval, i.e., 11.18 Hz and 12.66 Hz. The number of valid bootstrap samples is 176.

**S1 Fig. Histogram of axial first‑order equivalent natural frequency from bootstrap sampling**

**S1.2 Bending vibration**

Statistical results obtained from residual bootstrap based on small‑displacement dataset. The 95% confidence intervals are calculated by the percentile method. Number of valid samples after removing diverged identification cases: 176.

**S2 Table Statistical results of improved Bouc‑Wen parameters and first‑order equivalent natural frequency from residual‑bootstrap**

| **parameter** | **symbol** | **Original identification value** | **bootstrap means** | **standard deviation** | **Lower 95% confidence limit** | **Upper 95% confidence limit** |
| --- | --- | --- | --- | --- | --- | --- |
| Hysteresis evolution coefficient | $\rho$ | 1.6241 | 1.6473 | 0.1824 | 1.2812 | 1.9845 |
| Hysteresis shape coefficient | $\sigma$ | 3.1247 | 3.0862 | 0.2741 | 2.5336 | 3.6124 |
| hardening exponent | u | 1.4362 | 1.4127 | 0.1143 | 1.1964 | 1.6271 |
| Bending tangential stiffness of small displacement | $k_{E1}/\left( N\cdot mm^{-1} \right)$ | 7.4700 | 7.5124 | 0.4261 | 6.9235 | 8.1426 |
| Hysteresis amplification coefficient | $k_{A0}/N$ | 42.6317 | 43.1045 | 3.7244 | 35.8124 | 50.4731 |
| Bending first-order equivalent natural frequency | $f_{n}/\mathrm{Hz}$ | **13.20** | **13.26** | **0.43** | **12.41** | **14.12** |

Histogram of the first‑order equivalent natural frequency obtained from bootstrap sampling. The red vertical line represents the original identified value$f_{n}=13.20\mathrm{Hz}$. Two green dashed lines denote the bounds of the 95% confidence interval, i.e., 12.41Hz and 14.12 Hz. The number of valid bootstrap samples is 176.

**S2 Fig. Histogram of first‑order equivalent natural frequency from bootstrap sampling**
