# Supplementary material for: Equivalent linearization modeling and experimental validation of natural frequency and vibration isolation performance in metal braided hoses
Source: PLoS One. 2026 Nov 3;21(11):e0361869. doi: 10.1371/journal.pone.0361869 (PMC13730378; doi:10.1371/journal.pone.0361869)
Supplement: Supplementary file 2 [file pone.0361869.s002.docx]

**S2 Sensitivity analysis for model parameters and equivalent damping assumption**

To answer the question of how predicted natural frequency and vibration‑isolation results respond to variations of improved Bouc‑Wen parameters and the equivalent‑damping hypothesis, a two‑layer sensitivity analysis is performed: global parameter sensitivity based on existing bootstrap samples, plus local perturbation sensitivity for the equivalent‑damping assumption.

**S2.1 Global sensitivity using bootstrap samples**

We reuse 176 valid parameter sets obtained from residual‑bootstrap uncertainty quantification. Pearson correlation coefficients are computed between each model parameter and two outputs: (1) small‑displacement equivalent natural frequency $f_{n}$; (2) acceleration-transmission ratio $T_{A}$ under an experimentally‑validated low‑frequency small‑amplitude working point.

The small‑displacement natural frequency is dominated exclusively by the elastic tangent‑stiffness parameter $k_{E1}$, showing a strong positive correlation. Hysteresis‑related parameters ($\rho,\sigma,u,k_{A0}$) have negligible correlation with $f_{n}$. Physically, under infinitesimal displacement, the normalized hysteresis internal state $z\to0$; the hysteresis force contribution vanishes, so these parameters barely affect the origin tangent stiffness and natural frequency.

Nevertheless, hysteresis parameters strongly govern the area of the hysteresis loop and cyclic dissipated energy; hence, they exhibit moderate‑to‑strong correlation with equivalent damping and further with vibration‑isolation force‑transmission ratio $T_{F}$. Results are listed in S3 Table and S4 Table, as well as S3 Fig.(a).

**S3 Table Sensitivity analysis of each parameter along the axial direction**

| Symbols | Correlation coefficient with the axial natural frequency | Correlation coefficient with vibration isolation transmission rate |
| --- | --- | --- |
| $\rho$ | 0.082 | 0.441 |
| $\sigma$ | -0.114 | 0.523 |
| u | -0.076 | 0.476 |
| $k_{E1}$ | 0.962 | 0.327 |
| $k_{A0}$ | -0.091 | 0.618 |

**S4 Table Sensitivity analysis of each parameter along the bending direction**

| Symbols | Correlation coefficient with the bending natural frequency | Correlation coefficient with vibration isolation transmission rate |
| --- | --- | --- |
| $\rho$ | 0.074 | 0.412 |
| $\sigma$ | -0.103 | 0.497 |
| u | -0.068 | 0.443 |
| $k_{E1}$ | **0.957** | 0.301 |
| $k_{A0}$ | -0.084 | 0.584 |

**S3 Fig. Sensitivity summary chart**

**S2.2 Local sensitivity of equivalent‑damping assumption**

The equivalent viscous damping $C_{\mathrm{eq}}$ is obtained from the energy‑equality principle over one full hysteresis cycle, which is the core idealized assumption of equivalent linearization. To quantify the uncertainty introduced by this hypothesis, we apply $\boldsymbol{\pm}10\%$ relative perturbation to the baseline equivalent‑damping value, then recalculate the acceleration‑transmission ratio $T_{A}$ and the acceleration vibration‑level difference. For the validated low‑frequency small‑amplitude condition, $\pm10\%$ perturbation of equivalent damping leads to about $\pm0.8\sim0.9 \mathrm{dB}$ change in vibration‑level difference. This indicates that vibration isolation prediction has moderate sensitivity to the equivalent‑damping assumption; by contrast, natural frequency calculation is completely insensitive to damping.

**S5 Table Equivalent Damping Relative variation of vibration isolation transmission rate under disturbance**

| working condition | Baseline  $T_{A0}$ | Baseline  $L_{va0}$  (dB) | $C_{eq}\times0.9$  （‑10%）$T_{A1}$ | $L_{va1}$(dB) | $C_{eq}\times1.1$  （+10%）$T_{A2}$ | $L_{va2}$(dB) |
| --- | --- | --- | --- | --- | --- | --- |
| Axial vibration isolation | 0.2435 | 12.27 | 0.2679 | 11.44 | 0.2192 | 13.19 |
| Bending vibration isolation | 0.0752 | 22.48 | 0.0827 | 21.65 | 0.0677 | 23.39 |

**S2.3 Discussion on extrapolation uncertainty outside validated domain**

The equivalent‑linearization method is validated only for small‑amplitude, low‑frequency experimental conditions in this paper. Under large‑amplitude excitation, significant stiffness hardening occurs; at high frequency, potential rate‑dependent hysteresis effects may appear. Since there are no corresponding test data to validate the model in these regimes, model outputs for large‑amplitude or high‑frequency conditions cannot be trusted, and direct extrapolation should be avoided. This belongs to epistemic model uncertainty instead of stochastic measurement noise uncertainty.
