# Supplementary material for: Equivalent linearization modeling and experimental validation of natural frequency and vibration isolation performance in metal braided hoses
Source: PLoS One. 2026 Nov 3;21(11):e0361869. doi: 10.1371/journal.pone.0361869 (PMC13730378; doi:10.1371/journal.pone.0361869)
Supplement: Supplementary file 3 [file pone.0361869.s003.docx]

**S3 Measurement uncertainty for measured vibration‑isolation quantities**

S3.1 Method

The uncertainty of the vibration‑isolation index originates from accelerometer accuracy, test‑bench electrical noise, and dispersion from repeated tests of identical specimens. Combined standard uncertainty is calculated according to GUM (Guide to the expression of uncertainty in measurement). Expanded uncertainty is reported with a coverage factor $k=2$ (approximately 95 % confidence level).

**S6 Table Expanded measurement uncertainty (k=2) for vibration‑isolation transmission ratio**

| **Quantity** | **Measured value** | **Expanded uncertainty (**$k=2$**)** |
| --- | --- | --- |
| Axial acceleration vibration‑level difference $L_{va}$ | 12.27 dB | $\pm0.56\mathrm{dB}$ |
| Bending acceleration vibration‑level difference $L_{va}$ | 22.48 dB | $\pm0.56\mathrm{dB}$ |

S3.2 Note on conclusion generalizability

The trend of hose diameter, length, and braiding layers on acceleration vibration‑level difference is derived from limited specimens. These conclusions are only applicable to the specimen batch and parameter ranges investigated in this manuscript. Generalization to significantly different manufacturing processes, materials, or extreme dimension ranges is not recommended.
